# Supplementary figures and images for: Filamin protects myofibrils from contractile damage through changes in its mechanosensory region
Source: PLoS Genet. 2024 Jun 21;20(6):e1011101. doi: 10.1371/journal.pgen.1011101 (PMC11221683; doi:10.1371/journal.pgen.1011101)

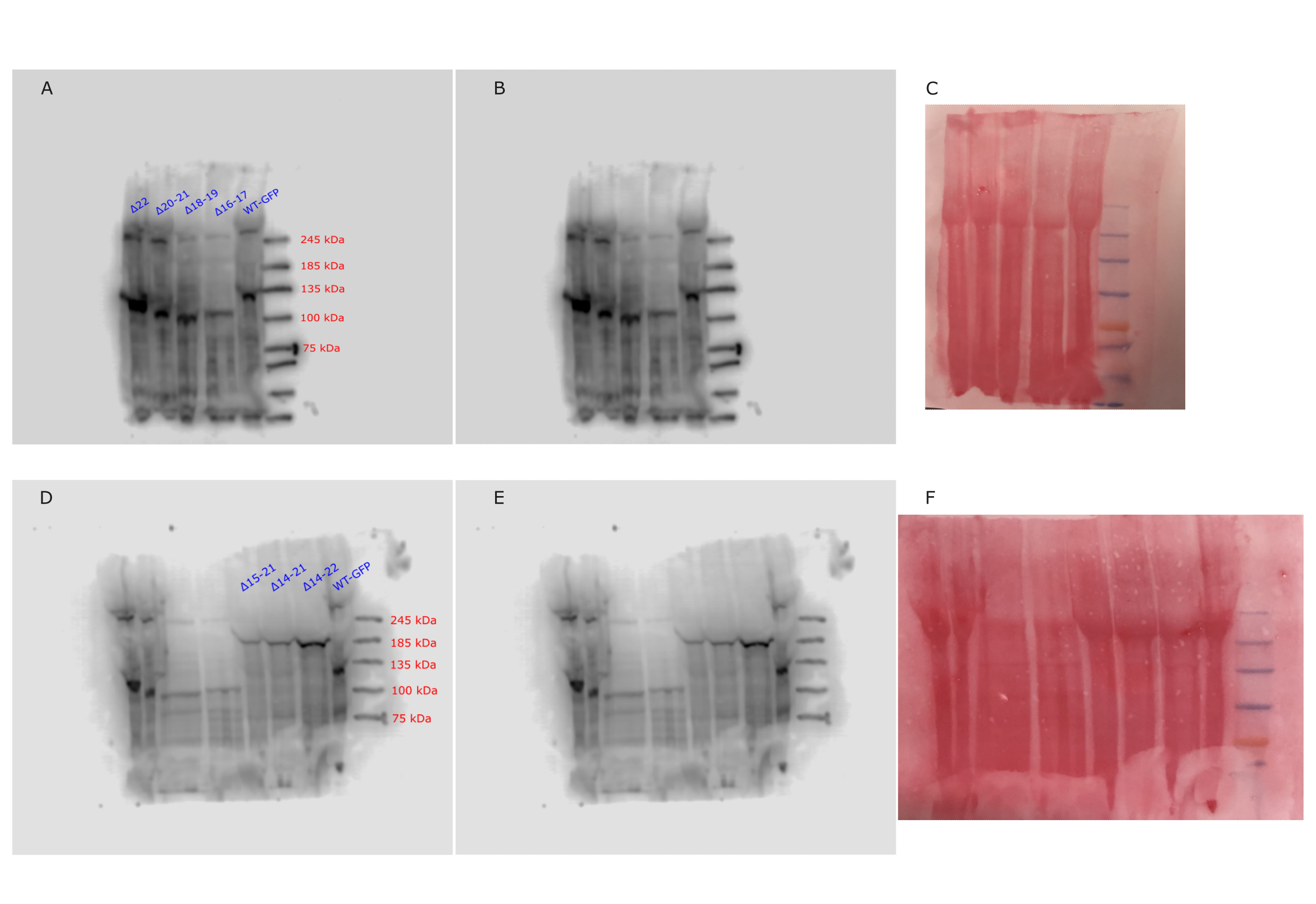

Supplement: S2 Fig — A and B) Original western blot images corresponding to Fig 3C left panel. C) Ponceau staining corresponding to Fig 3C left panel. D-E) Original western blot images corresponding to Fig 3C right panel. F) Ponceau staining corresponding to Fig 3C right panel. The molecular weights and the genotypes are annotated in panels A and D. (TIFF) [file pgen.1011101.s002.tiff]

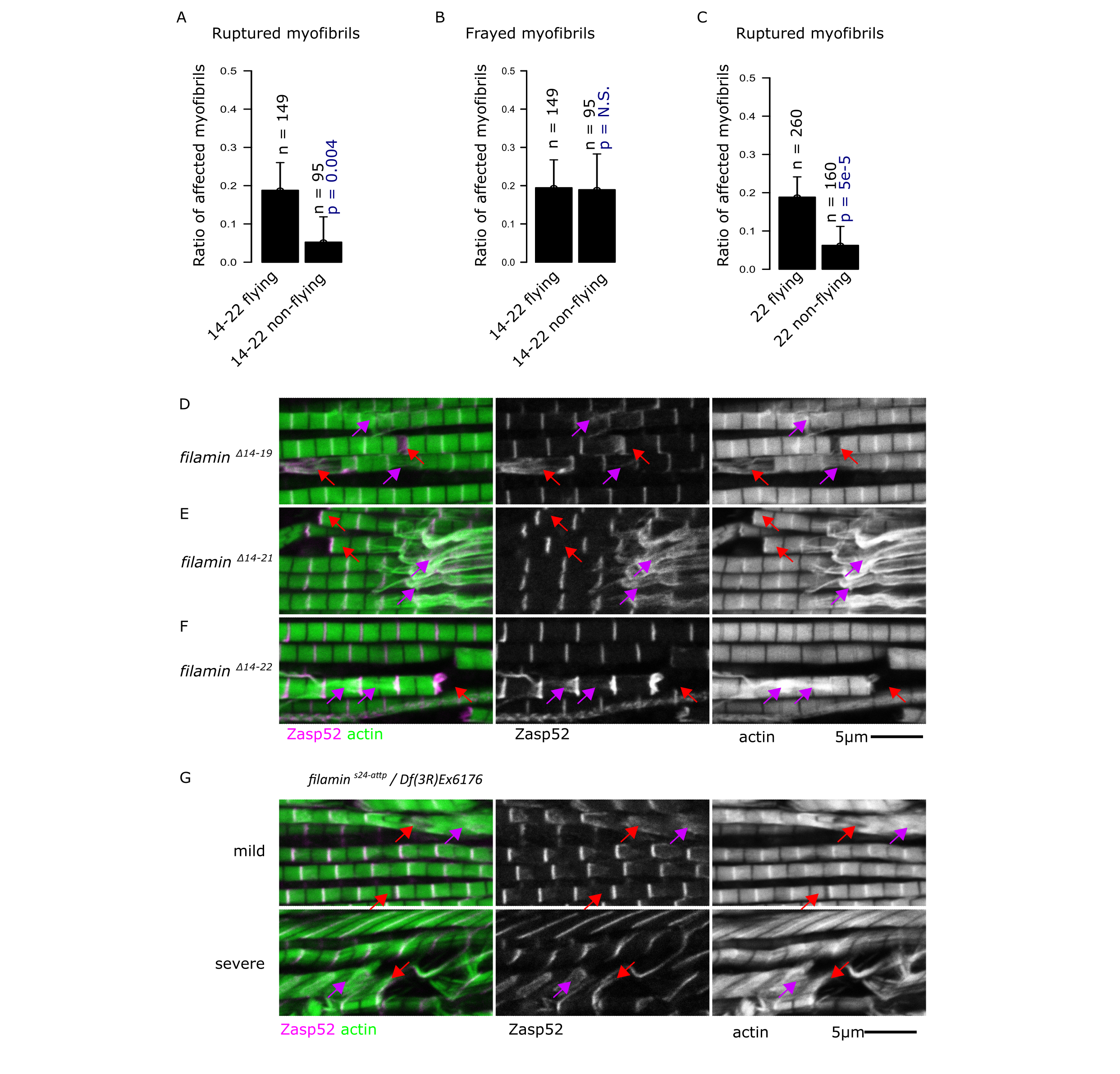

Supplement: S3 Fig — A-B) Plots of the phenotype ratios observed in the filaminΔ14-22-GFP mutant muscles in flying and non-flying conditions. A) The ratio of ruptured myofibrils decreased significantly in the non-flying condition. B) The ratio of frayed myofibrils over the total number of myofibrils does not change in the non-flying condition. C) The ratio of ruptured myofibrils over the total number of myofibrils in filaminΔ22-GFP mutant decreased significantly in the non-flying condition. Confidence intervals were calculated at 95 by an exact binomial test, and p-values were calculated using a 2-sample test for equality of proportions with continuity correction. D-F) Confocal image of filaminΔ14-19-GFP, filaminΔ14-21-GFP, and filaminΔ14–22 mutants over the Df(3R)Ex6176 deletion shows myofibrils that are both ruptured (red arrows) and frayed (purple arrows). G) The filaminΔs24-attp landing site mutant has a strong IFM phenotype. The top panel exhibits a mild phenotype characterized by ruptured (red arrows) and frayed myofibrils (purple arrows), while the lower panel displays a severe phenotype with similar features. In D-G, actin staining is in green, and Zasp52-mCherry is in magenta. The scale bar represents 5 μm. (TIFF) [file pgen.1011101.s003.tiff]

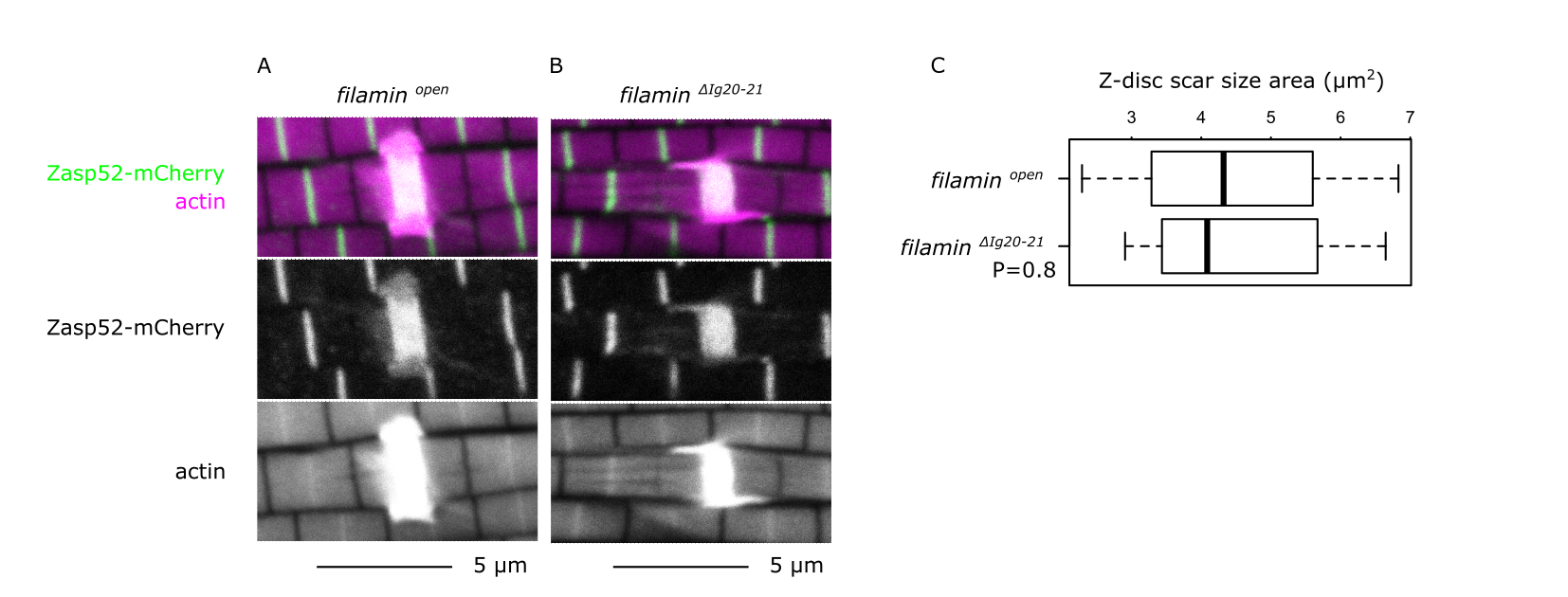

Supplement: S4 Fig — A) Confocal image of filaminopen-GFP mutant muscles with Zasp52-mCherry to mark the Z-disc in green and actin stained with phalloidin in magenta. B) Confocal image of filaminΔ20-21-GFP mutant muscles with Zasp52-mCherry to mark the Z-disc in green and actin stained with phalloidin in magenta. C) Boxplot of the measured area of enlarged Z-discs in both mutants. Welch two sample t-test was used for comparing the samples. (TIFF) [file pgen.1011101.s004.tiff]

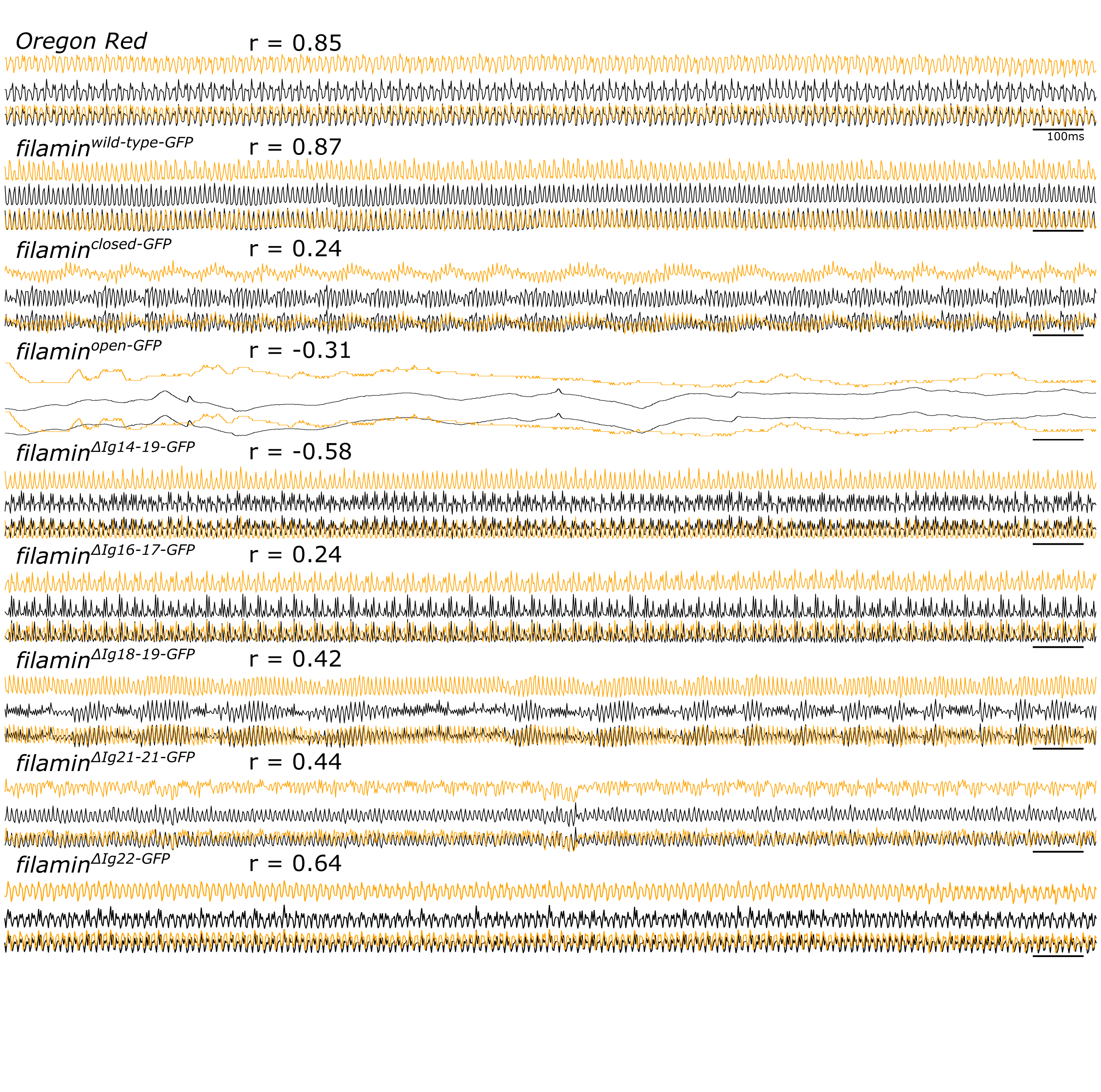

Supplement: S5 Fig — Scale bar in all is 100 ms. The right-wing recording is colored black. The left-wing recording is colored orange. The overlap plot is shown, and the correlation R values are noted. (TIFF) [file pgen.1011101.s005.tiff]
