# Supplementary material for: Filamin protects myofibrils from contractile damage through changes in its mechanosensory region
Source: PLoS Genet. 2024 Jun 21;20(6):e1011101. doi: 10.1371/journal.pgen.1011101 (PMC11221683; doi:10.1371/journal.pgen.1011101)
Supplement: S1 Data — (DOCX) [file pgen.1011101.s006.docx]

Supplementary information

Full genotypes of individuals used in each figure and panel

Figure 1

1. NA
2. NA
3. NA
4. *Zasp52^M02988-mCherry^ ; filamin^WT^* / *Df(3R)Ex6176*
5. *Zasp52^M02988-mCherry^ ; filamin^WT-GFP^* / *Df(3R)Ex6176*
6. *Zasp52^M02988-mCherry^ ; filamin^closed-GFP^* / *Df(3R)Ex6176*
7. *Zasp52^M02988-mCherry^ ; filamin^open-GFP^* / *Df(3R)Ex6176*
8. *Oregon Red, filamin^WT/WT^, filamin^WT-GFP/WT-GFP^, filamin^open/open^ , filamin^closed/closed^*
9. *Oregon Red, filamin^WT/WT^, filamin^WT-GFP/WT-GFP^, filamin^open/open^ , filamin^closed/closed^*
10. *Oregon Red, filamin^WT^ /+, filamin^WT-GFP/^/+, filamin^open^/+, filamin^closed^/+*

Figure 2

1. *Zasp52^M02988-mCherry^ ; filamin^closed-GFP^* / *Df(3R)Ex6176*
2. *Zasp52^M02988-mCherry^ ; filamin^open-GFP^* / *Df(3R)Ex6176*

Figure 3 – panel C

- *filamin^Δ22-GFP/Δ22-GFP^*
- *filamin^Δ20-21-GFP/Δ20-21-GFP^*
- *filamin^Δ18/19-GFP/Δ18-19-GFP^*
- *filamin^Δ16-17-GFP/Δ16-17-GFP^*
- *filamin^WT-GFP/WT-GFP^*
- *filamin^Δ15-21-GFP/Δ15-21-GFP^*
- *filamin^Δ14-12-GFP/Δ14-12-GFP^*
- *filamin^Δ14-22-GFP/Δ14-22-GFP^*
- *filamin^WT-GFP/WT-GFP^*

Figure 4

1. *Zasp52^M02988-mCherry^ ; filamin^WT-GFP^* / *Df(3R)Ex6176*
2. *Zasp52^M02988-mCherry^ ; filamin^Δ14-22^* / *Df(3R)Ex6176*
3. *Zasp52^M02988-mCherry^ ; filamin^Δ14-22^* / *Df(3R)Ex6176*
4. *Zasp52^M02988-mCherry^ ; filamin^Δ14-19^* / *Df(3R)Ex6176*
5. *Zasp52^M02988-mCherry^ ; filamin^Δ14-19^* / *Df(3R)Ex6176*
6. *Zasp52^M02988-mCherry^ ; filamin^Δ15-21^* / *Df(3R)Ex6176*

Figure 5

1. *Zasp52^M02988-mCherry^ ; filamin^WT-GFP^* / *Df(3R)Ex6176*
2. *Zasp52^M02988-mCherry^ ; filamin^Δ22^* / *Df(3R)Ex6176*
3. *Zasp52^M02988-mCherry^ ; filamin^Δ16-17^* / *Df(3R)Ex6176*
4. *Zasp52^M02988-mCherry^ ; filamin^Δ18-19^* / *Df(3R)Ex6176*
5. *Zasp52^M02988-mCherry^ ; filamin^Δ20-21^* / *Df(3R)Ex6176*

Figure 6

1. *filamin^WT-GFP/WT-GFP^*
2. *filamin^closed/closed^*
3. *filamin^closed/closed^*
4. *filamin^open/open^*
5. *filamin^Δ14-19/Δ14-19^*
6. *filamin^Δ14-22/Δ14-22^*

Figure 7

1. NA
2. *Oregon Red and filamin^Δ14-19/Δ14-19^*
3. *Oregon Red*
4. *filamin^WT-GFP/WT-GFP^*
5. *filamin^Δ14-19/Δ14-19^*
6. *filamin^open/open^*
7. *filamin^closed/closed^*
8. *filamin^Δ22/Δ22^*
9. *filamin^Δ16-17/Δ16-17^*
10. *filamin^Δ18-19/Δ18-19^*
11. *filamin^Δ20-21/Δ20-21^*
12. The following genotypes: *Oregon Red*, *filamin^WT-GFP/WT-GFP^*, *filamin^closed/closed^*, *filamin^Δ18-19/Δ18-19^*, *filamin^Δ22/Δ22^*, *filamin^Δ20-21/Δ20-2^*, *filamin^Δ14-19/Δ14-19^*, *filamin^Δ16-17/Δ16-17^*

Table. Summary of phenotypes in all genetic conditions analyzed.

| Mutation | Myofibril phenotype | Flight phenotype |
| --- | --- | --- |
| Oregon Red | Normal | Synchronized |
| *filamin^WT^* | Normal | Synchronized |
| *filamin^WT-GFP^* | Normal | Synchronized |
| *filamin^Δ14-19^* | Ruptured Z-discs and fraying | Unsynchronized |
| *filamin^Δ14-22^* | Ruptured Z-discs and fraying | Unsynchronized |
| *filamin^Δ14-21^* | Ruptured Z-discs and fraying | Unsynchronized |
| *filamin^Δ15-21^* | Ruptured Z-discs | Unsynchronized |
| *filamin^open^* | Enlarged Z-discs | Flightless |
| *filamin^closed^* | Ruptured Z-discs | Unsynchronized |
| *filamin^Δ22^* | Ruptured Z-discs | Unsynchronized |
| *filamin^Δ16-17^* | Ruptured Z-discs | Unsynchronized |
| *filamin^Δ18-19^* | Ruptured Z-discs | Unsynchronized |
| *filamin^Δ20-21^* | Enlarged Z-discs | Unsynchronized |
| *Mef2-Gal4, UAS-filamin-RNAi* | Ruptured Z-discs and fraying | Flightless |
