# Supplementary material for: Filamin protects myofibrils from contractile damage through changes in its mechanosensory region
Source: PLoS Genet. 2024 Jun 21;20(6):e1011101. doi: 10.1371/journal.pgen.1011101 (PMC11221683; doi:10.1371/journal.pgen.1011101)
Supplement: S2 Data — (PDF) [file pgen.1011101.s007.pdf]

Formatted Alignments

```
                                      10         20         30         40         50         60         70         80
Cher240_WT-{AAF55390}-20+2_lgs  MEAERDLAEDAQWKKIQQNTFTRWANEHLKTIDRSINNLETDLSDGLRLIALIEVLSQKRMPKYNKRPTFRSQKLENVSV
Cher240_WT_GFP
Cher240_closed_GFP
Cher240_open_GFP
Cher240_Dlg16-17_GFP
Cher240_Dlg18-19_GFP
Cher240_Dlg20-21_GFP
Cher240_Dlg22_GFP
Cher240_Dlg14-19_GFP
Cher240_Dlg15-21_GFP
Cher240_Dlg14-21_GFP
Cher240_Dlg14-22_GFP

                                      90        100        110        120        130        140        150        160
Cher240_WT-{AAF55390}-20+2_lgs  ALKFLQDEGIKIVNIDSSDIVDCKLKLILGLIWTLILHYSISMPMWDGEDDKQLNGSGHTPKQRLLNWIHAKIPDLPINN
Cher240_WT_GFP                  --------------------------------------------------------------------------------
Cher240_closed_GFP              --------------------------------------------------------------------------------
Cher240_open_GFP                --------------------------------------------------------------------------------
Cher240_Dlg16-17_GFP            --------------------------------------------------------------------------------
Cher240_Dlg18-19_GFP            --------------------------------------------------------------------------------
Cher240_Dlg20-21_GFP            --------------------------------------------------------------------------------
Cher240_Dlg22_GFP               --------------------------------------------------------------------------------
Cher240_Dlg14-19_GFP            --------------------------------------------------------------------------------
Cher240_Dlg15-21_GFP            --------------------------------------------------------------------------------
Cher240_Dlg14-21_GFP            --------------------------------------------------------------------------------
Cher240_Dlg14-22_GFP            --------------------------------------------------------------------------------
```

```
                                       170       180       190       200       210       220       230       240
Cher240_WT-{AAF55390}-20+2_Igs  FTNDWTTGKAVGALVDACAPGLCPDWELWDPKDAVQNASEAMGLADDWLNVRQLIKPEELVNPNVDEQSMMTYLSQYPNS
Cher240_WT_GFP                  --------------------------------------------------------------------------------
Cher240_closed_GFP              --------------------------------------------------------------------------------
Cher240_open_GFP                --------------------------------------------------------------------------------
Cher240_DIg16-17_GFP            --------------------------------------------------------------------------------
Cher240_DIg18-19_GFP            --------------------------------------------------------------------------------
Cher240_DIg20-21_GFP            --------------------------------------------------------------------------------
Cher240_DIg22_GFP               --------------------------------------------------------------------------------
Cher240_DIg14-19_GFP            --------------------------------------------------------------------------------
Cher240_DIg15-21_GFP            --------------------------------------------------------------------------------
Cher240_DIg14-21_GFP            --------------------------------------------------------------------------------
Cher240_DIg14-22_GFP            --------------------------------------------------------------------------------

                                       250       260       270       280       290       300       310       320
Cher240_WT-{AAF55390}-20+2_Igs  KLKTGAPLRPKTNPNRVRAYGPGIEPIGPVVGAPANFTVETFSAGKGSVDVDIQGPNGEIEKADVRFNNDKNLTYTVSYI
Cher240_WT_GFP                  --------------------------------------------------------------------------------
Cher240_closed_GFP              --------------------------------------------------------------------------------
Cher240_open_GFP                --------------------------------------------------------------------------------
Cher240_DIg16-17_GFP            --------------------------------------------------------------------------------
Cher240_DIg18-19_GFP            --------------------------------------------------------------------------------
Cher240_DIg20-21_GFP            --------------------------------------------------------------------------------
Cher240_DIg22_GFP               --------------------------------------------------------------------------------
Cher240_DIg14-19_GFP            --------------------------------------------------------------------------------
Cher240_DIg15-21_GFP            --------------------------------------------------------------------------------
Cher240_DIg14-21_GFP            --------------------------------------------------------------------------------
Cher240_DIg14-22_GFP            --------------------------------------------------------------------------------

                                       330       340       350       360       370       380       390       400
Cher240_WT-{AAF55390}-20+2_Igs  PKSEGSHKVAVKFSGRDIPKSPFPVKVEGHAGDASKVKVTGPGIQPNGVTIKKPTFFDILAKDAGRGVPEVIIIDPANHK
Cher240_WT_GFP                  --------------------------------------------------------------------------------
Cher240_closed_GFP              --------------------------------------------------------------------------------
Cher240_open_GFP                --------------------------------------------------------------------------------
Cher240_DIg16-17_GFP            --------------------------------------------------------------------------------
Cher240_DIg18-19_GFP            --------------------------------------------------------------------------------
Cher240_DIg20-21_GFP            --------------------------------------------------------------------------------
Cher240_DIg22_GFP               --------------------------------------------------------------------------------
Cher240_DIg14-19_GFP            --------------------------------------------------------------------------------
Cher240_DIg15-21_GFP            --------------------------------------------------------------------------------
Cher240_DIg14-21_GFP            --------------------------------------------------------------------------------
Cher240_DIg14-22_GFP            --------------------------------------------------------------------------------
```

```
                                         410       420       430       440       450       460       470       480
Cher240_WT-{AAF55390}-20+2_Igs  TSVAAKVRQLENDTWRCEYVTALQGLHSVNVFYAGTPIPNSPFPVKVAPLSDARKVRASGRGLQATGVRVGDDADFKIYT
Cher240_WT_GFP                  --------------------------------------------------------------------------------
Cher240_closed_GFP              --------------------------------------------------------------------------------
Cher240_open_GFP                --------------------------------------------------------------------------------
Cher240_DIg16-17_GFP            --------------------------------------------------------------------------------
Cher240_DIg18-19_GFP            --------------------------------------------------------------------------------
Cher240_DIg20-21_GFP            --------------------------------------------------------------------------------
Cher240_DIg22_GFP               --------------------------------------------------------------------------------
Cher240_DIg14-19_GFP            --------------------------------------------------------------------------------
Cher240_DIg15-21_GFP            --------------------------------------------------------------------------------
Cher240_DIg14-21_GFP            --------------------------------------------------------------------------------
Cher240_DIg14-22_GFP            --------------------------------------------------------------------------------

                                         490       500       510       520       530       540       550       560
Cher240_WT-{AAF55390}-20+2_Igs  EGAGEGEPEVRVIGPGGMNQNVMQSKVDGNTYECHYYPTKEGRYVIMVTFAGQEVAKSPFEVKVGPKKESSIVAYGPGLS
Cher240_WT_GFP                  --------------------------------------------------------------------------------
Cher240_closed_GFP              --------------------------------------------------------------------------------
Cher240_open_GFP                --------------------------------------------------------------------------------
Cher240_DIg16-17_GFP            --------------------------------------------------------------------------------
Cher240_DIg18-19_GFP            --------------------------------------------------------------------------------
Cher240_DIg20-21_GFP            --------------------------------------------------------------------------------
Cher240_DIg22_GFP               --------------------------------------------------------------------------------
Cher240_DIg14-19_GFP            --------------------------------------------------------------------------------
Cher240_DIg15-21_GFP            --------------------------------------------------------------------------------
Cher240_DIg14-21_GFP            --------------------------------------------------------------------------------
Cher240_DIg14-22_GFP            --------------------------------------------------------------------------------

                                         570       580       590       600       610       620       630       640
Cher240_WT-{AAF55390}-20+2_Igs  SGVIGYPAAFVVETNGETGALGFTVAGPSQAEIECHDNGDGSALVKYHPTAVGEYAVHILCDNEDIPKSPFIAQILPRTD
Cher240_WT_GFP                  --------------------------------------------------------------------------------
Cher240_closed_GFP              --------------------------------------------------------------------------------
Cher240_open_GFP                --------------------------------------------------------------------------------
Cher240_DIg16-17_GFP            --------------------------------------------------------------------------------
Cher240_DIg18-19_GFP            --------------------------------------------------------------------------------
Cher240_DIg20-21_GFP            --------------------------------------------------------------------------------
Cher240_DIg22_GFP               --------------------------------------------------------------------------------
Cher240_DIg14-19_GFP            --------------------------------------------------------------------------------
Cher240_DIg15-21_GFP            --------------------------------------------------------------------------------
Cher240_DIg14-21_GFP            --------------------------------------------------------------------------------
Cher240_DIg14-22_GFP            --------------------------------------------------------------------------------
```

```
                                                  650       660       670       680       690       700       710       720
Cher240_WT-{AAF55390}-20+2_Igs  FHPELVKASGPGLEKNGVTINQPTSFTVDPSKAGNAPLDVVVQDVFGTKLPVELKNNPDGTKKVTYTPTSGVPHTVEVNY
Cher240_WT_GFP                  --------------------------------------------------------------------------------
Cher240_closed_GFP              --------------------------------------------------------------------------------
Cher240_open_GFP                --------------------------------------------------------------------------------
Cher240_DIg16-17_GFP            --------------------------------------------------------------------------------
Cher240_DIg18-19_GFP            --------------------------------------------------------------------------------
Cher240_DIg20-21_GFP            --------------------------------------------------------------------------------
Cher240_DIg22_GFP               --------------------------------------------------------------------------------
Cher240_DIg14-19_GFP            --------------------------------------------------------------------------------
Cher240_DIg15-21_GFP            --------------------------------------------------------------------------------
Cher240_DIg14-21_GFP            --------------------------------------------------------------------------------
Cher240_DIg14-22_GFP            --------------------------------------------------------------------------------

                                                  730       740       750       760       770       780       790       800
Cher240_WT-{AAF55390}-20+2_Igs  GGVSTPNSPHRVYVGVPVDAAKVQAFGPWLQPGVRPNAATHFNVDAREAGDAELKVKIIHEETKIEVPCRIIDNEDNTYS
Cher240_WT_GFP                  --------------------------------------------------------------------------------
Cher240_closed_GFP              --------------------------------------------------------------------------------
Cher240_open_GFP                --------------------------------------------------------------------------------
Cher240_DIg16-17_GFP            --------------------------------------------------------------------------------
Cher240_DIg18-19_GFP            --------------------------------------------------------------------------------
Cher240_DIg20-21_GFP            --------------------------------------------------------------------------------
Cher240_DIg22_GFP               --------------------------------------------------------------------------------
Cher240_DIg14-19_GFP            --------------------------------------------------------------------------------
Cher240_DIg15-21_GFP            --------------------------------------------------------------------------------
Cher240_DIg14-21_GFP            --------------------------------------------------------------------------------
Cher240_DIg14-22_GFP            --------------------------------------------------------------------------------

                                                  810       820       830       840       850       860       870       880
Cher240_WT-{AAF55390}-20+2_Igs  VEVIPPSKGAYTTTMTYGGQRVPLGEKVVVEQTVDVSKIKVDGLEPTAPLNSLQQFRIITHGLPKADLAVTITSPSGNRI
Cher240_WT_GFP                  --------------------------------------------------------------------------------
Cher240_closed_GFP              --------------------------------------------------------------------------------
Cher240_open_GFP                --------------------------------------------------------------------------------
Cher240_DIg16-17_GFP            --------------------------------------------------------------------------------
Cher240_DIg18-19_GFP            --------------------------------------------------------------------------------
Cher240_DIg20-21_GFP            --------------------------------------------------------------------------------
Cher240_DIg22_GFP               --------------------------------------------------------------------------------
Cher240_DIg14-19_GFP            --------------------------------------------------------------------------------
Cher240_DIg15-21_GFP            --------------------------------------------------------------------------------
Cher240_DIg14-21_GFP            --------------------------------------------------------------------------------
Cher240_DIg14-22_GFP            --------------------------------------------------------------------------------
```

```
                                                 890       900       910       920       930       940       950       960
Cher240_WT-{AAF55390}-20+2_Igs  KAHIIPTAEGFLVNFTPTQLGEYLLSICFGGTPITPRPFRLQCLTGSDSNKVQAFGPGLERGIVGQPAEFMIDTRGAGQG
Cher240_WT_GFP                  --------------------------------------------------------------------------------
Cher240_closed_GFP              --------------------------------------------------------------------------------
Cher240_open_GFP                --------------------------------------------------------------------------------
Cher240_DIg16-17_GFP            --------------------------------------------------------------------------------
Cher240_DIg18-19_GFP            --------------------------------------------------------------------------------
Cher240_DIg20-21_GFP            --------------------------------------------------------------------------------
Cher240_DIg22_GFP               --------------------------------------------------------------------------------
Cher240_DIg14-19_GFP            --------------------------------------------------------------------------------
Cher240_DIg15-21_GFP            --------------------------------------------------------------------------------
Cher240_DIg14-21_GFP            --------------------------------------------------------------------------------
Cher240_DIg14-22_GFP            --------------------------------------------------------------------------------
```

```
                                                 970       980       990      1000      1010      1020      1030      1040
Cher240_WT-{AAF55390}-20+2_Igs  GLGVTVEGPCEAAINCRDNGDGTCNVAYLPTEAGDYTVNITFNERHITGSPFQPLIVPVPNLKNTRVSGIGIQPHGVIMN
Cher240_WT_GFP                  --------------------------------------------------------------------------------
Cher240_closed_GFP              --------------------------------------------------------------------------------
Cher240_open_GFP                --------------------------------------------------------------------------------
Cher240_DIg16-17_GFP            --------------------------------------------------------------------------------
Cher240_DIg18-19_GFP            --------------------------------------------------------------------------------
Cher240_DIg20-21_GFP            --------------------------------------------------------------------------------
Cher240_DIg22_GFP               --------------------------------------------------------------------------------
Cher240_DIg14-19_GFP            --------------------------------------------------------------------------------
Cher240_DIg15-21_GFP            --------------------------------------------------------------------------------
Cher240_DIg14-21_GFP            --------------------------------------------------------------------------------
Cher240_DIg14-22_GFP            --------------------------------------------------------------------------------
```

```
                                                1050      1060      1070      1080      1090      1100      1110      1120
Cher240_WT-{AAF55390}-20+2_Igs  AATDFMVDMSKVGSNIDSGKLSCAIFDPMGHVLPSKIVQGPTDDIFRIMYTPFEAGRHTIELMYDNIPVPGSPFVVNVKS
Cher240_WT_GFP                  --------------------------------------------------------------------------------
Cher240_closed_GFP              --------------------------------------------------------------------------------
Cher240_open_GFP                --------------------------------------------------------------------------------
Cher240_DIg16-17_GFP            --------------------------------------------------------------------------------
Cher240_DIg18-19_GFP            --------------------------------------------------------------------------------
Cher240_DIg20-21_GFP            --------------------------------------------------------------------------------
Cher240_DIg22_GFP               --------------------------------------------------------------------------------
Cher240_DIg14-19_GFP            --------------------------------------------------------------------------------
Cher240_DIg15-21_GFP            --------------------------------------------------------------------------------
Cher240_DIg14-21_GFP            --------------------------------------------------------------------------------
Cher240_DIg14-22_GFP            --------------------------------------------------------------------------------
```

	1130 1140 1150 1160 1170 1180 1190 1200
Cher240_WT-{AAF55390}-20+2_Igs	GCDPARCKAYGPGLEKGLTNQKNKFTVETKGAGNGGLSLAIEGPSEAKMTCTDNRDGSCDVDYLATDPGEYDITIRFADK
Cher240_WT_GFP	--
Cher240_closed_GFP	--
Cher240_open_GFP	--
Cher240_DIg16-17_GFP	--
Cher240_DIg18-19_GFP	--
Cher240_DIg20-21_GFP	--
Cher240_DIg22_GFP	--
Cher240_DIg14-19_GFP	--
Cher240_DIg15-21_GFP	--
Cher240_DIg14-21_GFP	--
Cher240_DIg14-22_GFP	--

	1210 1220 1230 1240 1250 1260 1270 1280
Cher240_WT-{AAF55390}-20+2_Igs	HIPGSPFRVLVEETVDPSKVKVYGPGIEHGQVRESVPTFFNVDVGEAGPGRIAVKLTNSEGIPVDNLRVEDKGNCIYAVH
Cher240_WT_GFP	--
Cher240_closed_GFP	--
Cher240_open_GFP	--
Cher240_DIg16-17_GFP	--
Cher240_DIg18-19_GFP	--
Cher240_DIg20-21_GFP	--
Cher240_DIg22_GFP	--
Cher240_DIg14-19_GFP	--
Cher240_DIg15-21_GFP	--
Cher240_DIg14-21_GFP	--
Cher240_DIg14-22_GFP	--

	1290 1300 1310 1320 1330 1340 1350 1360
Cher240_WT-{AAF55390}-20+2_Igs	YVPPKAGSVLTCQVKFSEVEVPCSPFVMTVFPKSEPTKVKVKGVNEKKKTPASLPAEFEIDTKQAGQADINVAIKNPKGK
Cher240_WT_GFP	--
Cher240_closed_GFP	--
Cher240_open_GFP	--
Cher240_DIg16-17_GFP	--
Cher240_DIg18-19_GFP	--
Cher240_DIg20-21_GFP	--
Cher240_DIg22_GFP	--
Cher240_DIg14-19_GFP	--
Cher240_DIg15-21_GFP	--
Cher240_DIg14-21_GFP	--
Cher240_DIg14-22_GFP	--

```
                                    1370      1380      1390      1400      1410      1420      1430      1440
Cher240_WT-{AAF55390}-20+2_Igs  AMQPRLEEVSTGTYVVSFVPDECGTYQCSIKYGDKEIEGSPFKLEAFPTGEAKKCKLVEQAPKIQTSGSQSHLKVDAREA
Cher240_WT_GFP                  --------------------------------------------------------------------------------
Cher240_closed_GFP              --------------------------------------------------------------------------------
Cher240_open_GFP                --------------------------------------------------------------------------------
Cher240_Dlg16-17_GFP            --------------------------------------------------------------------------------
Cher240_Dlg18-19_GFP            --------------------------------------------------------------------------------
Cher240_Dlg20-21_GFP            --------------------------------------------------------------------------------
Cher240_Dlg22_GFP               --------------------------------------------------------------------------------
Cher240_Dlg14-19_GFP            --------------------------------------------------------------------------------
Cher240_Dlg15-21_GFP            --------------------------------------------------------------------------------
Cher240_Dlg14-21_GFP            --------------------------------------------------------------------------------
Cher240_Dlg14-22_GFP            --------------------------------------------------------------------------------

                                    1450      1460      1470      1480      1490      1500      1510      1520
Cher240_WT-{AAF55390}-20+2_Igs  GDGAVTCKITNKAGSEIVDIDVIEKDGFFDILYALNDPGDYDINVKFGGKDIPNGSFSIKAVESIEQYSHSEYIEEHTTK
Cher240_WT_GFP                  --------------------------------------------------------------------------------
Cher240_closed_GFP              --------------------------------------------------------------------------------
Cher240_open_GFP                --------------------------------------------------------------------------------
Cher240_Dlg16-17_GFP            --------------------------------------------------------------------------------
Cher240_Dlg18-19_GFP            --------------------------------------------------------------------------------
Cher240_Dlg20-21_GFP            --------------------------------------------------------------------------------
Cher240_Dlg22_GFP               --------------------------------------------------------------------------------
Cher240_Dlg14-19_GFP            --------------------------------------------------------------------------------
Cher240_Dlg15-21_GFP            --------------------------------------------------------------------------------
Cher240_Dlg14-21_GFP            --------------------------------------------------------------------------------
Cher240_Dlg14-22_GFP            --------------------------------------------------------------------------------

                                    1530      1540      1550      1560      1570      1580      1590      1600
Cher240_WT-{AAF55390}-20+2_Igs  VVQQTTQSELVNGKSEITYRSVAFEKLPLPTTGGNVTAEVRMPSGKVDKPVIQDNRDGTVSVKYDPREEGSHELVVKYNG
Cher240_WT_GFP                  --------------------------------------EVRMPSGKVDKPVIQDNRDGTVSVKYDPREEGSHELVVKYNG
Cher240_closed_GFP              --------------------------------------EVRMPSGKVDKPVIQDNRDGTVSVKYDPREEGSHELVVKYNG
Cher240_open_GFP                --------------------------------------EVRMPSGKVDKPVIQDNRDGTVSVKYDPREEGSHELVVKYNG
Cher240_Dlg16-17_GFP            --------------------------------------EVRMPSGKVDKPVIQDNRDGTVSVKYDPREEGSHELVVKYNG
Cher240_Dlg18-19_GFP            --------------------------------------EVRMPSGKVDKPVIQDNRDGTVSVKYDPREEGSHELVVKYNG
Cher240_Dlg20-21_GFP            --------------------------------------EVRMPSGKVDKPVIQDNRDGTVSVKYDPREEGSHELVVKYNG
Cher240_Dlg22_GFP               --------------------------------------EVRMPSGKVDKPVIQDNRDGTVSVKYDPREEGSHELVVKYNG
Cher240_Dlg14-19_GFP            --------------------------------------------------------------------------------
Cher240_Dlg15-21_GFP            --------------------------------------EVRMPSGKVDKPVIQDNRDGTVSVKYDPREEGSHELVVKYNG
Cher240_Dlg14-21_GFP            --------------------------------------------------------------------------------
Cher240_Dlg14-22_GFP            --------------------------------------------------------------------------------
```

```
                                            1610      1620      1630      1640      1650      1660      1670      1680
Cher240_WT-{AAF55390}-20+2_lgs    EPVQGSPFKFHVDSITSGYVTAYGPGLTHGVTGEPANFTISTKGASAGGLTMAVEGPSKADINYHDNKDGTVSVQYLPTA
Cher240_WT_GFP                    EPVQGSPFKFHVDSITSGYVTAYGPGLTHGVTGEPANFTISTKGASAGGLTMAVEGPSKADINYHDNKDGTVSVQYLPTA
Cher240_closed_GFP                EPVQGSPFKFHVDSITSGYVTAYGPGLTHGVTGEPANFTISTKGASAGGLTMAVEGPSKADINYHDNKDGTVSVQYLPTA
Cher240_open_GFP                  EPVQGSPFKFHVDSITSGYVTAYGPGLTHGVTGEPANFTISTKGASAGGLTMAVEGPSKADINYHDNKDGTVSVQYLPTA
Cher240_Dlg16-17_GFP              EPVQGSPFKFHVDSITSGYVTAYGPGLTHGVTGEPANFTISTKGASAGGLTMAVEGPSKADINYHDNKDGTVSVQYLPTA
Cher240_Dlg18-19_GFP              EPVQGSPFKFHVDSITSGYVTAYGPGLTHGVTGEPANFTISTKGASAGGLTMAVEGPSKADINYHDNKDGTVSVQYLPTA
Cher240_Dlg20-21_GFP              EPVQGSPFKFHVDSITSGYVTAYGPGLTHGVTGEPANFTISTKGASAGGLTMAVEGPSKADINYHDNKDGTVSVQYLPTA
Cher240_Dlg22_GFP                 EPVQGSPFKFHVDSITSGYVTAYGPGLTHGVTGEPANFTISTKGASAGGLTMAVEGPSKADINYHDNKDGTVSVQYLPTA
Cher240_Dlg14-19_GFP              --------------------------------------------------------------------------------
Cher240_Dlg15-21_GFP              EPVQGSPFKFHVDSITSG--------------------------------------------------------------
Cher240_Dlg14-21_GFP              --------------------------------------------------------------------------------
Cher240_Dlg14-22_GFP              --------------------------------------------------------------------------------

                                            1690      1700      1710      1720      1730      1740      1750      1760
Cher240_WT-{AAF55390}-20+2_lgs    PGEYQVSVRFGDKHIKGSPYFAKITGEGRKRNQISVGSCSEVTMPGDITDDDLRALNASIQAPSGLEEPCFLKRMPTGNI
Cher240_WT_GFP                    PGEYQVSVRFGDKHIKGSPYFAKITGEGRKRNQISVGSCSEVTMPGDITDDDLRALNASIQAPSGLEEPCFLKRMPTGNI
Cher240_closed_GFP                PGEYQVSVRFGDKHIKGSPYFAKITGEGRFRSSLFLGSCSEVTMPGDITDDDLRALNASIQAPSGLEEPCFLKRMPTGNI
Cher240_open_GFP                  PGEYQVSVRFGDKHIKGSPYFAKITGEGRKRNQESVGSCSEVTMPGDITDDDLRALNASIQAPSGLEEPCFLKRMPTGNI
Cher240_Dlg16-17_GFP              PGEYQVSVRFGDKHIKGSPYFAKITGEG----------------------------------------------------
Cher240_Dlg18-19_GFP              PGEYQVSVRFGDKHIKGSPYFAKITGEGRKRNQISVGSCSEVTMPGDITDDDLRALNASIQAPSGLEEPCFLKRMPTGNI
Cher240_Dlg20-21_GFP              PGEYQVSVRFGDKHIKGSPYFAKITGEGRKRNQISVGSCSEVTMPGDITDDDLRALNASIQAPSGLEEPCFLKRMPTGNI
Cher240_Dlg22_GFP                 PGEYQVSVRFGDKHIKGSPYFAKITGEGRKRNQISVGSCSEVTMPGDITDDDLRALNASIQAPSGLEEPCFLKRMPTGNI
Cher240_Dlg14-19_GFP              --------------------------------------------------------------------------------
Cher240_Dlg15-21_GFP              --------------------------------------------------------------------------------
Cher240_Dlg14-21_GFP              --------------------------------------------------------------------------------
Cher240_Dlg14-22_GFP              --------------------------------------------------------------------------------

                                            1770      1780      1790      1800      1810      1820      1830      1840
Cher240_WT-{AAF55390}-20+2_lgs    GISFTPREIGEHLVSVKRLGKHINNSPFKVTVCEREVGDAKKVKVSGTGLKEGQTHADNIFSVDTRNAGFGGLSVSIEGP
Cher240_WT_GFP                    GISFTPREIGEHLVSVKRLGKHINNSPFKVTVCEREVGDAKKVKVSGTGLKEGQTHADNIFSVDTRNAGFGGLSVSIEGP
Cher240_closed_GFP                GISFTPREIGEHLVSVKRLGKHINNSPFKVTVCEREVGDAKKVKVSGTGLKEGQTHADNIFSVDTRNAGFGGLSVSIEGP
Cher240_open_GFP                  GISFTPREIGEHLVSVKRLGKHINNSPFKVTVCEREVGDAKKVKVSGTGLKEGQTHADNIFSVDTRNAGFGGLSVSIEGP
Cher240_Dlg16-17_GFP              --------------------------------------------------------------------------------
Cher240_Dlg18-19_GFP              GISFTPREIGEHLVSVKRLGKHINNSPFKVTVCEREVGDAKKVKVSGTGLKEGQTHADNIFSVDTRNAGFGGLSVSIEGP
Cher240_Dlg20-21_GFP              GISFTPREIGEHLVSVKRLGKHINNSPFKVTVCEREVGDAKKVKVSGTGLKEGQTHADNIFSVDTRNAGFGGLSVSIEGP
Cher240_Dlg22_GFP                 GISFTPREIGEHLVSVKRLGKHINNSPFKVTVCEREVGDAKKVKVSGTGLKEGQTHADNIFSVDTRNAGFGGLSVSIEGP
Cher240_Dlg14-19_GFP              --------------------------------------------------------------------------------
Cher240_Dlg15-21_GFP              --------------------------------------------------------------------------------
Cher240_Dlg14-21_GFP              --------------------------------------------------------------------------------
Cher240_Dlg14-22_GFP              --------------------------------------------------------------------------------
```

```
                                   1850      1860      1870      1880      1890      1900      1910      1920
Cher240_WT-{AAF55390}-20+2_lgs     SKAEIQCTDKDDGTLNISYKPTEPGYYIVNLKFADHHVEGSPFTVKVAGEGSNRKREKIQRERDAVPITEIGSQCKLTFK
Cher240_WT_GFP                     SKAEIQCTDKDDGTLNISYKPTEPGYYIVNLKFADHHVEGSPFTVKVAGEGSNRKREKIQRERDAVPITEIGSQCKLTFK
Cher240_closed_GFP                 SKAEIQCTDKDDGTLNISYKPTEPGYYIVNLKFADHHVEGSPFTVKVAGEGSNRFRSSLFLERDAVPITEIGSQCKLTFK
Cher240_open_GFP                   SKAEIQCTDKDDGTLNISYKPTEPGYYIVNLKFADHHVEGSPFTVKVAGEGSNRKREKEQRERDAVPITEIGSQCKLTFK
Cher240_Dlg16-17_GFP               ---------------------------------------------------SNRKREKIQRERDAVPITEIGSQCKLTFK
Cher240_Dlg18-19_GFP               SKAEIQCTDKDDGTLNISYKPTEPGYYIVNLKFADHHVEGSPFTVKVAGEGSN---------------------------
Cher240_Dlg20-21_GFP               SKAEIQCTDKDDGTLNISYKPTEPGYYIVNLKFADHHVEGSPFTVKVAGEGSNRKREKIQRERDAVPITEIGSQCKLTFK
Cher240_Dlg22_GFP                  SKAEIQCTDKDDGTLNISYKPTEPGYYIVNLKFADHHVEGSPFTVKVAGEGSNRKREKIQRERDAVPITEIGSQCKLTFK
Cher240_Dlg14-19_GFP               --------------------------------------------------------------------------------
Cher240_Dlg15-21_GFP               --------------------------------------------------------------------------------
Cher240_Dlg14-21_GFP               --------------------------------------------------------------------------------
Cher240_Dlg14-22_GFP               --------------------------------------------------------------------------------

                                   1930      1940      1950      1960      1970      1980      1990      2000
Cher240_WT-{AAF55390}-20+2_lgs     MPGITSFDLAACVTSPSNVTEDAEIQEVEDGLYAVHFVPKELGVHTVSVRYSEMHIPGSPFQFTVGPLRDSGSHLVKAGG
Cher240_WT_GFP                     MPGITSFDLAACVTSPSNVTEDAEIQEVEDGLYAVHFVPKELGVHTVSVRYSEMHIPGSPFQFTVGPLRDSGSHLVKAGG
Cher240_closed_GFP                 MPGITSFDLAACVTSPSNVTEDAEIQEVEDGLYAVHFVPKELGVHTVSVRYSEMHIPGSPFQFTVGPLRDSGSHLVKAGG
Cher240_open_GFP                   MPGITSFDLAACVTSPSNVTEDAEIQEVEDGLYAVHFVPKELGVHTVSVRYSEMHIPGSPFQFTVGPLRDSGSHLVKAGG
Cher240_Dlg16-17_GFP               MPGITSFDLAACVTSPSNVTEDAEIQEVEDGLYAVHFVPKELGVHTVSVRYSEMHIPGSPFQFTVGPLRDSGSHLVKAGG
Cher240_Dlg18-19_GFP               --------------------------------------------------------------------------------
Cher240_Dlg20-21_GFP               MPGITSFDLAACVTSPSNVTEDAEIQEVEDGLYAVHFVPKELGVHTVSVRYSEMHIPGSPFQFTVGPLRDSGSHLVKAGG
Cher240_Dlg22_GFP                  MPGITSFDLAACVTSPSNVTEDAEIQEVEDGLYAVHFVPKELGVHTVSVRYSEMHIPGSPFQFTVGPLRDSGSHLVKAGG
Cher240_Dlg14-19_GFP               --------------------------------------------------------------------------------
Cher240_Dlg15-21_GFP               --------------------------------------------------------------------------------
Cher240_Dlg14-21_GFP               --------------------------------------------------------------------------------
Cher240_Dlg14-22_GFP               --------------------------------------------------------------------------------

                                   2010      2020      2030      2040      2050      2060      2070      2080
Cher240_WT-{AAF55390}-20+2_lgs     SGLERGVVGEAAEFNVWTREAGGGSLAISVEGPSKADIEFKDRKDGSCDVSYKVTEPGEYRVGLKFNDRHIPDSPFKVYV
Cher240_WT_GFP                     SGLERGVVGEAAEFNVWTREAGGGSLAISVEGPSKADIEFKDRKDGSCDVSYKVTEPGEYRVGLKFNDRHIPDSPFKVYV
Cher240_closed_GFP                 SGLERGVVGEAAEFNVWTREAGGGSLAISVEGPSKADIEFKDRKDGSCDVSYKVTEPGEYRVGLKFNDRHIPDSPFKVYV
Cher240_open_GFP                   SGLERGVVGEAAEFNVWTREAGGGSLAISVEGPSKADIEFKDRKDGSCDVSYKVTEPGEYRVGLKFNDRHIPDSPFKVYV
Cher240_Dlg16-17_GFP               SGLERGVVGEAAEFNVWTREAGGGSLAISVEGPSKADIEFKDRKDGSCDVSYKVTEPGEYRVGLKFNDRHIPDSPFKVYV
Cher240_Dlg18-19_GFP               --------------------------------------------------------------------------------
Cher240_Dlg20-21_GFP               SGLERGVVGEAAEFNVWTREAGGGSLAISVEGPSKADIEFKDRKDGSCDVSYKVTEPGEYRVGLKFNDRHIPDSPFKVYV
Cher240_Dlg22_GFP                  SGLERGVVGEAAEFNVWTREAGGGSLAISVEGPSKADIEFKDRKDGSCDVSYKVTEPGEYRVGLKFNDRHIPDSPFKVYV
Cher240_Dlg14-19_GFP               --------------------------------------------------------------------------------
Cher240_Dlg15-21_GFP               --------------------------------------------------------------------------------
Cher240_Dlg14-21_GFP               --------------------------------------------------------------------------------
Cher240_Dlg14-22_GFP               --------------------------------------------------------------------------------
```

```
                                 2090      2100      2110      2120      2130      2140      2150      2160
Cher240_WT-{AAF55390}-20+2_lgs   SPDAGDAHKLEVQQFPQGNIQADAPYQFMVRKNGAKGELDAKIVAPSGTDDDCFIQVIDGEMYSVRFYPRENGIHAIHVK
Cher240_WT_GFP                   SPDAGDAHKLEVQQFPQGNIQADAPYQFMVRKNGAKGELDAKIVAPSGTDDDCFIQVIDGEMYSVRFYPRENGIHAIHVK
Cher240_closed_GFP               SPDAGDAHKLEVQQFPQGNIQADAPYQFMVRKNGAKGELDAKIVAPSGTDDDCFIQVIDGEMYSVRFYPRENGIHAIHVK
Cher240_open_GFP                 SPDAGDAHKLEVQQFPQGNIQADAPYQFMVRKNGAKGELDAKIVAPSGTDDDCFIQVIDGEMYSVRFYPRENGIHAIHVK
Cher240_Dlg16-17_GFP             SPDAGDAHKLEVQQFPQGNIQADAPYQFMVRKNGAKGELDAKIVAPSGTDDDCFIQVIDGEMYSVRFYPRENGIHAIHVK
Cher240_Dlg18-19_GFP             --DAGDAHKLEVQQFPQGNIQADAPYQFMVRKNGAKGELDAKIVAPSGTDDDCFIQVIDGEMYSVRFYPRENGIHAIHVK
Cher240_Dlg20-21_GFP             SP------------------------------------------------------------------------------
Cher240_Dlg22_GFP                SPDAGDAHKLEVQQFPQGNIQADAPYQFMVRKNGAKGELDAKIVAPSGTDDDCFIQVIDGEMYSVRFYPRENGIHAIHVK
Cher240_Dlg14-19_GFP             -----DAHKLEVQQFPQGNIQADAPYQFMVRKNGAKGELDAKIVAPSGTDDDCFIQVIDGEMYSVRFYPRENGIHAIHVK
Cher240_Dlg15-21_GFP             --------------------------------------------------------------------------------
Cher240_Dlg14-21_GFP             --------------------------------------------------------------------------------
Cher240_Dlg14-22_GFP             --------------------------------------------------------------------------------

                                 2170      2180      2190      2200      2210      2220      2230      2240
Cher240_WT-{AAF55390}-20+2_lgs   FNGVHIPDSPFRIKVGKDVADPAAVHASGNGLDEVKTGHKADFIINTCNAGVGTLAVSIDGPSKVAMDCTEVEEGYKVRY
Cher240_WT_GFP                   FNGVHIPDSPFRIKVGKDVADPAAVHASGNGLDEVKTGHKADFIINTCNAGVGTLAVSIDGPSKVAMDCTEVEEGYKVRY
Cher240_closed_GFP               FNGVHIPDSPFRIKVGKDVADPAAVHASGNGLDEVKTGHKADFIINTCNAGVGTLAVSIDGPSKVAMDCTEVEEGYKVRY
Cher240_open_GFP                 FNGVHIPDSPFRIKVGKDVADPAAVHASGNGLDEVKTGHKADFIINTCNAGVGTLAVSIDGPSKVAMDCTEVEEGYKVRY
Cher240_Dlg16-17_GFP             FNGVHIPDSPFRIKVGKDVADPAAVHASGNGLDEVKTGHKADFIINTCNAGVGTLAVSIDGPSKVAMDCTEVEEGYKVRY
Cher240_Dlg18-19_GFP             FNGVHIPDSPFRIKVGKDVADPAAVHASGNGLDEVKTGHKADFIINTCNAGVGTLAVSIDGPSKVAMDCTEVEEGYKVRY
Cher240_Dlg20-21_GFP             --------------------------------------------------------------------------------
Cher240_Dlg22_GFP                FNGVHIPDSPFRIKVGKDVADPAAVHASGNGLDEVKTGHKADFIINTCNAGVGTLAVSIDGPSKVAMDCTEVEEGYKVRY
Cher240_Dlg14-19_GFP             FNGVHIPDSPFRIKVGKDVADPAAVHASGNGLDEVKTGHKADFIINTCNAGVGTLAVSIDGPSKVAMDCTEVEEGYKVRY
Cher240_Dlg15-21_GFP             --------------------------------------------------------------------------------
Cher240_Dlg14-21_GFP             --------------------------------------------------------------------------------
Cher240_Dlg14-22_GFP             --------------------------------------------------------------------------------

                                 2250      2260      2270      2280      2290      2300      2310      2320
Cher240_WT-{AAF55390}-20+2_lgs   TPLLPGEHYITVKYNNMHIVGSPFKVNATGDKLADEGAQETSTVIVETVQKVAKGGKNTGVHLPTFKSDASKVVSKGMGL
Cher240_WT_GFP                   TPLLPGEHYITVKYNNMHIVGSPFKVNATGDKLADEGAQETSTVIVETVQKVAKGGKNTGVHLPTFKSDASKVVSKGMGL
Cher240_closed_GFP               TPLLPGEHYITVKYNNMHIVGSPFKVNATGDKLADEGAQETSTVIVETVQKVAKGGKNTGVHLPTFKSDASKVVSKGMGL
Cher240_open_GFP                 TPLLPGEHYITVKYNNMHIVGSPFKVNATGDKLADEGAQETSTVIVETVQKVAKGGKNTGVHLPTFKSDASKVVSKGMGL
Cher240_Dlg16-17_GFP             TPLLPGEHYITVKYNNMHIVGSPFKVNATGDKLADEGAQETSTVIVETVQKVAKGGKNTGVHLPTFKSDASKVVSKGMGL
Cher240_Dlg18-19_GFP             TPLLPGEHYITVKYNNMHIVGSPFKVNATGDKLADEGAQETSTVIVETVQKVAKGGKNTGVHLPTFKSDASKVVSKGMGL
Cher240_Dlg20-21_GFP             ------------------------------DKLADEGAQETSTVIVETVQKVAKGGKNTGVHLPTFKSDASKVVSKGMGL
Cher240_Dlg22_GFP                TPLLPGEHYITVKYNNMHIVGSPFKVNATGDKLADEGAQETSTVIVETVQKVAKGGKNTGVHLPTFKSD-----------
Cher240_Dlg14-19_GFP             TPLLPGEHYITVKYNNMHIVGSPFKVNATGDKLADEGAQETSTVIVETVQKVAKGGKNTGVHLPTFKSDASKVVSKGMGL
Cher240_Dlg15-21_GFP             ------------------------------DKLADEGAQETSTVIVETVQKVAKGGKNTGVHLPTFKSDASKVVSKGMGL
Cher240_Dlg14-21_GFP             ------------------------------DKLADEGAQETSTVIVETVQKVAKGGKNTGVHLPTFKSDASKVVSKGMGL
Cher240_Dlg14-22_GFP             --------------------------------------------------------------------------------
```

```
                                   2330      2340      2350      2360      2370      2380      2390      2400
Cher240_WT-{AAF55390}-20+2_lgs     KKAYIGKQNQFSISATDAGNNILYVGMYGPKGPCEEFHVKHAGHNNYNVQYLVRDRGQYVLLIKWGEEHIPGSPFQIDV-
Cher240_WT_GFP                     KKAYIGKQNQFSISATDAGNNILYVGMYGPKGPCEEFHVKHAGHNNYNVQYLVRDRGQYVLLIKWGEEHIPGSPFQIDVS
Cher240_closed_GFP                 KKAYIGKQNQFSISATDAGNNILYVGMYGPKGPCEEFHVKHAGHNNYNVQYLVRDRGQYVLLIKWGEEHIPGSPFQIDVS
Cher240_open_GFP                   KKAYIGKQNQFSISATDAGNNILYVGMYGPKGPCEEFHVKHAGHNNYNVQYLVRDRGQYVLLIKWGEEHIPGSPFQIDVS
Cher240_Dlg16-17_GFP               KKAYIGKQNQFSISATDAGNNILYVGMYGPKGPCEEFHVKHAGHNNYNVQYLVRDRGQYVLLIKWGEEHIPGSPFQIDVS
Cher240_Dlg18-19_GFP               KKAYIGKQNQFSISATDAGNNILYVGMYGPKGPCEEFHVKHAGHNNYNVQYLVRDRGQYVLLIKWGEEHIPGSPFQIDVS
Cher240_Dlg20-21_GFP               KKAYIGKQNQFSISATDAGNNILYVGMYGPKGPCEEFHVKHAGHNNYNVQYLVRDRGQYVLLIKWGEEHIPGSPFQIDVS
Cher240_Dlg22_GFP                  -------------------------------------------------------------------------------S
Cher240_Dlg14-19_GFP               KKAYIGKQNQFSISATDAGNNILYVGMYGPKGPCEEFHVKHAGHNNYNVQYLVRDRGQYVLLIKWGEEHIPGSPFQIDVS
Cher240_Dlg15-21_GFP               KKAYIGKQNQFSISATDAGNNILYVGMYGPKGPCEEFHVKHAGHNNYNVQYLVRDRGQYVLLIKWGEEHIPGSPFQIDVS
Cher240_Dlg14-21_GFP               KKAYIGKQNQFSISATDAGNNILYVGMYGPKGPCEEFHVKHAGHNNYNVQYLVRDRGQYVLLIKWGEEHIPGSPFQIDVS
Cher240_Dlg14-22_GFP               -------------------------------------------------------------------------------S
```

```
                                   2410      2420      2430      2440      2450      2460      2470      2480
Cher240_WT-{AAF55390}-20+2_lgs     --------------------------------------------------------------------------------
Cher240_WT_GFP                     SSSMSKGEELFTGVVPILVELDGDVNGHKFSVSGEGEGDATYGKLTLKFICTTGKLPVPWPTLVTTLTYGVQCFSRYPDH
Cher240_closed_GFP                 SSSMSKGEELFTGVVPILVELDGDVNGHKFSVSGEGEGDATYGKLTLKFICTTGKLPVPWPTLVTTLTYGVQCFSRYPDH
Cher240_open_GFP                   SSSMSKGEELFTGVVPILVELDGDVNGHKFSVSGEGEGDATYGKLTLKFICTTGKLPVPWPTLVTTLTYGVQCFSRYPDH
Cher240_Dlg16-17_GFP               SSSMSKGEELFTGVVPILVELDGDVNGHKFSVSGEGEGDATYGKLTLKFICTTGKLPVPWPTLVTTLTYGVQCFSRYPDH
Cher240_Dlg18-19_GFP               SSSMSKGEELFTGVVPILVELDGDVNGHKFSVSGEGEGDATYGKLTLKFICTTGKLPVPWPTLVTTLTYGVQCFSRYPDH
Cher240_Dlg20-21_GFP               SSSMSKGEELFTGVVPILVELDGDVNGHKFSVSGEGEGDATYGKLTLKFICTTGKLPVPWPTLVTTLTYGVQCFSRYPDH
Cher240_Dlg22_GFP                  SSSMSKGEELFTGVVPILVELDGDVNGHKFSVSGEGEGDATYGKLTLKFICTTGKLPVPWPTLVTTLTYGVQCFSRYPDH
Cher240_Dlg14-19_GFP               SSSMSKGEELFTGVVPILVELDGDVNGHKFSVSGEGEGDATYGKLTLKFICTTGKLPVPWPTLVTTLTYGVQCFSRYPDH
Cher240_Dlg15-21_GFP               SSSMSKGEELFTGVVPILVELDGDVNGHKFSVSGEGEGDATYGKLTLKFICTTGKLPVPWPTLVTTLTYGVQCFSRYPDH
Cher240_Dlg14-21_GFP               SSSMSKGEELFTGVVPILVELDGDVNGHKFSVSGEGEGDATYGKLTLKFICTTGKLPVPWPTLVTTLTYGVQCFSRYPDH
Cher240_Dlg14-22_GFP               SSSMSKGEELFTGVVPILVELDGDVNGHKFSVSGEGEGDATYGKLTLKFICTTGKLPVPWPTLVTTLTYGVQCFSRYPDH
```

```
                                   2490      2500      2510      2520      2530      2540      2550      2560
Cher240_WT-{AAF55390}-20+2_lgs     --------------------------------------------------------------------------------
Cher240_WT_GFP                     MKRHDFFKSAMPEGYVQERTIFFKDDGNYKTRAEVKFEGDTLVNRIELKGIDFKEDGNILGHKLEYNYNSHNVYIMADKQ
Cher240_closed_GFP                 MKRHDFFKSAMPEGYVQERTIFFKDDGNYKTRAEVKFEGDTLVNRIELKGIDFKEDGNILGHKLEYNYNSHNVYIMADKQ
Cher240_open_GFP                   MKRHDFFKSAMPEGYVQERTIFFKDDGNYKTRAEVKFEGDTLVNRIELKGIDFKEDGNILGHKLEYNYNSHNVYIMADKQ
Cher240_Dlg16-17_GFP               MKRHDFFKSAMPEGYVQERTIFFKDDGNYKTRAEVKFEGDTLVNRIELKGIDFKEDGNILGHKLEYNYNSHNVYIMADKQ
Cher240_Dlg18-19_GFP               MKRHDFFKSAMPEGYVQERTIFFKDDGNYKTRAEVKFEGDTLVNRIELKGIDFKEDGNILGHKLEYNYNSHNVYIMADKQ
Cher240_Dlg20-21_GFP               MKRHDFFKSAMPEGYVQERTIFFKDDGNYKTRAEVKFEGDTLVNRIELKGIDFKEDGNILGHKLEYNYNSHNVYIMADKQ
Cher240_Dlg22_GFP                  MKRHDFFKSAMPEGYVQERTIFFKDDGNYKTRAEVKFEGDTLVNRIELKGIDFKEDGNILGHKLEYNYNSHNVYIMADKQ
Cher240_Dlg14-19_GFP               MKRHDFFKSAMPEGYVQERTIFFKDDGNYKTRAEVKFEGDTLVNRIELKGIDFKEDGNILGHKLEYNYNSHNVYIMADKQ
Cher240_Dlg15-21_GFP               MKRHDFFKSAMPEGYVQERTIFFKDDGNYKTRAEVKFEGDTLVNRIELKGIDFKEDGNILGHKLEYNYNSHNVYIMADKQ
Cher240_Dlg14-21_GFP               MKRHDFFKSAMPEGYVQERTIFFKDDGNYKTRAEVKFEGDTLVNRIELKGIDFKEDGNILGHKLEYNYNSHNVYIMADKQ
Cher240_Dlg14-22_GFP               MKRHDFFKSAMPEGYVQERTIFFKDDGNYKTRAEVKFEGDTLVNRIELKGIDFKEDGNILGHKLEYNYNSHNVYIMADKQ
```

```
                                2570      2580      2590      2600      2610      2620      2630      2640
Cher240_WT-{AAF55390}-20+2_lgs  --------------------------------------------------------------------------------
Cher240_WT_GFP                  KNGIKANFKTRHNIEDGGVQLADHYQQNTPIGDGPVLLPDNHYLSTQSALSKDPNEKRDHMVLLEFVTAAGITHGMDELY
Cher240_closed_GFP              KNGIKANFKTRHNIEDGGVQLADHYQQNTPIGDGPVLLPDNHYLSTQSALSKDPNEKRDHMVLLEFVTAAGITHGMDELY
Cher240_open_GFP                KNGIKANFKTRHNIEDGGVQLADHYQQNTPIGDGPVLLPDNHYLSTQSALSKDPNEKRDHMVLLEFVTAAGITHGMDELY
Cher240_Dlg16-17_GFP            KNGIKANFKTRHNIEDGGVQLADHYQQNTPIGDGPVLLPDNHYLSTQSALSKDPNEKRDHMVLLEFVTAAGITHGMDELY
Cher240_Dlg18-19_GFP            KNGIKANFKTRHNIEDGGVQLADHYQQNTPIGDGPVLLPDNHYLSTQSALSKDPNEKRDHMVLLEFVTAAGITHGMDELY
Cher240_Dlg20-21_GFP            KNGIKANFKTRHNIEDGGVQLADHYQQNTPIGDGPVLLPDNHYLSTQSALSKDPNEKRDHMVLLEFVTAAGITHGMDELY
Cher240_Dlg22_GFP               KNGIKANFKTRHNIEDGGVQLADHYQQNTPIGDGPVLLPDNHYLSTQSALSKDPNEKRDHMVLLEFVTAAGITHGMDELY
Cher240_Dlg14-19_GFP            KNGIKANFKTRHNIEDGGVQLADHYQQNTPIGDGPVLLPDNHYLSTQSALSKDPNEKRDHMVLLEFVTAAGITHGMDELY
Cher240_Dlg15-21_GFP            KNGIKANFKTRHNIEDGGVQLADHYQQNTPIGDGPVLLPDNHYLSTQSALSKDPNEKRDHMVLLEFVTAAGITHGMDELY
Cher240_Dlg14-21_GFP            KNGIKANFKTRHNIEDGGVQLADHYQQNTPIGDGPVLLPDNHYLSTQSALSKDPNEKRDHMVLLEFVTAAGITHGMDELY
Cher240_Dlg14-22_GFP            KNGIKANFKTRHNIEDGGVQLADHYQQNTPIGDGPVLLPDNHYLSTQSALSKDPNEKRDHMVLLEFVTAAGITHGMDELY

Cher240_WT-{AAF55390}-20+2_lgs
Cher240_WT_GFP                  KG*
Cher240_closed_GFP              KG*
Cher240_open_GFP                KG*
Cher240_Dlg16-17_GFP            KG*
Cher240_Dlg18-19_GFP            KG*
Cher240_Dlg20-21_GFP            KG*
Cher240_Dlg22_GFP               KG*
Cher240_Dlg14-19_GFP            KG*
Cher240_Dlg15-21_GFP            KG*
Cher240_Dlg14-21_GFP            KG*
Cher240_Dlg14-22_GFP            KG*
```
