# Supplementary material for: Filamin protects myofibrils from contractile damage through changes in its mechanosensory region
Source: PLoS Genet. 2024 Jun 21;20(6):e1011101. doi: 10.1371/journal.pgen.1011101 (PMC11221683; doi:10.1371/journal.pgen.1011101)
Supplement: Attachment — Submitted filename: Response to reviewers.docx [file pgen.1011101.s009.docx]

Dear Dr Gonzalez Morales,

Thank you very much for submitting your Research Article entitled 'Filamin protects myofibrils from contractile damage through changes in its mechanosensory region' to PLOS Genetics.

The manuscript was fully evaluated at the editorial level and by independent peer reviewers. The reviewers appreciated the attention to an important problem, but raised some substantial concerns about the current manuscript. Based on the reviews, we will not be able to accept this version of the manuscript, but we would be willing to review a much-revised version. We cannot, of course, promise publication at that time.

Should you decide to revise the manuscript for further consideration here, your revisions should address the specific points made by each reviewer. We will also require a detailed list of your responses to the review comments and a description of the changes you have made in the manuscript.

If you decide to revise the manuscript for further consideration at PLOS Genetics, please aim to resubmit within the next 60 days, unless it will take extra time to address the concerns of the reviewers, in which case we would appreciate an expected resubmission date by email to plosgenetics@plos.org.

If present, accompanying reviewer attachments are included with this email; please notify the journal office if any appear to be missing. They will also be available for download from the link below. You can use this link to log into the system when you are ready to submit a revised version, having first consulted our [Submission Checklist](http://journals.plos.org/plosgenetics/s/submit-now#loc-submission-checklist).

To enhance the reproducibility of your results, we recommend that you deposit your laboratory protocols in protocols.io, where a protocol can be assigned its own identifier (DOI) such that it can be cited independently in the future. Additionally, PLOS ONE offers an option to publish peer-reviewed clinical study protocols. Read more information on sharing protocols at <https://plos.org/protocols?utm_medium=editorial-email&utm_source=authorletters&utm_campaign=protocols>

Please be aware that our [data availability policy](http://journals.plos.org/plosgenetics/s/data-availability) requires that all numerical data underlying graphs or summary statistics are included with the submission, and you will need to provide this upon resubmission if not already present. In addition, we do not permit the inclusion of phrases such as "data not shown" or "unpublished results" in manuscripts. All points should be backed up by data provided with the submission.

While revising your submission, please upload your figure files to the [Preflight Analysis and Conversion Engine](http://pace.apexcovantage.com/) (PACE) digital diagnostic tool.  PACE helps ensure that figures meet PLOS requirements. To use PACE, you must first register as a user. Then, login and navigate to the UPLOAD tab, where you will find detailed instructions on how to use the tool. If you encounter any issues or have any questions when using PACE, please email us at figures@plos.org.

PLOS has incorporated [Similarity Check](http://www.crossref.org/crosscheck.html), powered by iThenticate, into its journal-wide submission system in order to screen submitted content for originality before publication. Each PLOS journal undertakes screening on a proportion of submitted articles. You will be contacted if needed following the screening process.

To resubmit, use the link below and 'Revise Submission' in the 'Submissions Needing Revision' folder.

We are sorry that we cannot be more positive about your manuscript at this stage. Please do not hesitate to contact us if you have any concerns or questions.

Yours sincerely,

Pablo Wappner
Academic Editor
PLOS Genetics

Gregory P. Copenhaver
Editor-in-Chief
PLOS Genetics

**Reviewer's Responses to Questions**

**Reviewer #1:** This manuscript focuses on the role of filamin in Drosophila indirect flight muscle, using genetic and cell biological approaches. The confocal images are of high quality and the quantitative approach to studying the myofibril defects is appreciated. Further, the development of a device that independently assesses each wing movement is novel and yields interesting results. Overall, it is a nice study that provides new insights into the role of filamin in myofibril structure and function. There are a number of issues that should be addressed by the authors to improve the structure and content of the manuscript.

Major Points:

1. The abstract does not provide much detail as to what was done experimentally and how the conclusions were reached. Why was the C-terminal targeted? Is it the mechanosensory region? What is novel about the mutants? Why were closed vs. open mutants tested? Does one of them prevent/enhance elastic changes? I realize that brevity is necessary in the abstract, but the various points need to be logically connected.

We have changed the abstract to include more experimental details and better explain the logic behind the experiments.

2. Results: Since the analysis is done in hemizygous flies, the authors should comment that only a single copy of the gene is necessary for complete rescue (is this true in regard to flight ability?).

We have described in an earlier article that heterozygous loss of function filamin mutants fly as well as control flies (González-Morales N, et al, 2017 10.1371/journal.pgen.1006880). Back then, we did not use the open or closed alleles. We analyzed their flight ability and noticed that while the heterozygous closed mutants can fly as well as the controls, the heterozygous open mutants have a compromised flight ability. We have added the new data to Figure 1. We have made clear that a single copy of the filamin gene is enough for complete rescue.

What was the reason that mutations were tested over the deletion instead of as homozygotes? On line 281, the authors state “We analyzed these mutants over a deficiency background to rule out the effect of secondary background mutations.” By this, I suppose they mean recessive mutations that might be on the mutated chromosome. Can this be explained more thoroughly? Is this the general reasoning for the study being done over the deletion? Why mention it only on line 282?

We added a clarification in line 164 and then in line 238. We removed the mention in line 281

Line 164: “To standardize the genetic background and introduce a Z-disc fluorescence marker, we first generated a line carrying the *Df(3R)Ex6176* deficiency, which completely removes the filamin gene, and a *Zasp52-mCherry* allele that robustly labels the Z-disc. We crossed this line with the filamin mutants and analyzed the IFM of the progeny carrying a filamin mutant over *Df(3R)Ex6176* and a copy of Zasp52-mCherry allele.”

Line 281: We first analyzed the IFM phenotypes of the large deletion mutants over the *Df(3R)Ex6176* deficiency chromosome by confocal microscopy.

In addition, we added full genotype details as a supplementary data file

3. Results: Is it not unexpected that the filamin mutants with ruptured Z disks are able to fly? Quantitative flight tests seem an important missing element in the paper, but this is largely made up for by the novel wing beat analysis. Perhaps this should be mentioned earlier, i.e., the flight abilities are quantitatively assessed at the end of the Results.

It is not unexpected that filamin loss of function mutants can partially fly for the first 2 weeks after adult emergence because it has been reported previously by us (González-Morales N, et al, 2017 10.1371/journal.pgen.1006880) and by Nick Brown’s Lab. Green H. et al, 2018 10.7554/eLife.35783).

However, those reports did not use the open or closed mutants. We now added flight data in Figure 1.

4. Line 186: the eccentric contractions referred to here are in human muscles. Are you implying that you are testing for eccentric contractions as being causative in Drosophila? Is there evidence for eccentric contractions and resulting myofibril defects in Drosophila?

There is no evidence in Drosophila that eccentric and not concentric contractions result in myofibril rupture. We removed the mention of eccentric contractions and replaced it with muscle contraction in humans.

5. Figure 3C: The left panel may be problematic, as it appears to contain multiple blots spliced together.

Each panel represents a single blot; they are not spliced together. To eliminate confusion regarding the nature of the blots, we have included the raw images in the supplementary data. Considering that filamin is a large protein and is not abundantly present in the IFM, western blotting poses challenges. To put this into perspective, filamin transcripts constitute only about 2% of the highly abundant Z-disc protein Zasp52 (Spletter M. 2018 [https://doi.org/10.7554/eLife.34058 -- Supplementary File 1](https://doi.org/10.7554/eLife.34058%20--%20Supplementary%20File%201)).

6. The conclusion regarding the origin of enlarged Z-discs is speculative. See the following paper wherein multiple references are given for IFMs that display enlarged Z-discs under various genotypes/conditions: <https://doi.org/10.3390/biology11081137> . These other instances should at least be mentioned and/or argued to fit (or not fit) with the authors’ speculations.

We agree that discussing other conditions that lead to enlarged Z-discs is important. Our model is that a variety of mutations lead to myofibril hypercontraction which indirectly forces filamin into an open conformation. We did not add it originally because it is a highly speculative statement. However, we added a discussion paragraph to mention these other instances in which enlarged discs are observed.

“Similar enlarged Z-disc phenotypes like the one in filamin open mutants have been shown before from a diverse set of genetic conditions and sometimes are referred to as zebra bodies. For example, by affecting the tension generated by myofibril contraction through a mutation in the motor domain in the myosin heavy chain (MHC R249D; [44]), by reducing the amount of myosin-II, or its regulatory light chain [45], or by overexpressing truncated forms of the M-line protein Sals [46]. In addition, massively affecting transcription or translation of IFM-specific genes also leads to enlarged Z-discs [47,48]. A speculative scenario is that these conditions cause muscle hypercontraction, which indirectly results in filamin opening, therefore creating enlarged discs. Mutations in MHC indeed cause muscle hypercontraction [49].”

7. The localization of filamin in the various filamin mutants is interesting, but without quantification, can the authors really conclude that levels are reduced in the normal sarcomeres of mutants that have enlarged Z-discs? At the least, it would be helpful to state (assuming this is the case) that the confocal images in the mutants are taken at the same settings as the control.

We have remade Figure 6 to include detailed quantification measurements of filamin GFP in different mutants. Specifically, this figure now features a plot illustrating the decrease in filamin Z-disc localization in most filamin mutants (Figure 6G), another plot demonstrating the accumulation of filamin in various mutants leading to ruptured discs (Figure 6H), highlighting that the C-terminal part of filamin is not required for this accumulation. Additionally, there is a plot showing the accumulation of filamin in the open and 20-21 mutants (Figure 6I). Lastly, a plot demonstrates that in the open mutant, intact Z-discs in proximity to an enlarged Z-disc have less filamin than Z-discs not in proximity to an enlarged Z-disc (Figure 6J).

These measurements were possible because all the images were taken at the same settings as the controls. We have added a section in the methods section to explain the measurements and microscopy settings.

8. Line 394: “All these experimental data have led to the hypothesis that filamin senses mechanical damage at the Z-disc where it coordinates myofibril repair. Here we test this hypothesis…” I see no evidence in the manuscript that filamin coordinates myofibril repair or testing of this issue. In fact, it is unclear that such repair occurs in Drosophila IFM. The requirement of filamin to prevent Z-disc rupturing or myofibril fraying likely indicates it plays a role in maintaining stability, rather than in repairing the defects, i.e., “mechanoprotection”, as mentioned by the authors.

We agree that the usage of the term repair is misleading and is based only on the muscle phenotypes of filamin constitutively open and closed mutants. In addition, we don’t know how if at all repair happens in the IFM. We envision the repair as a system that “glues” the damaged and about-to-break Z-discs before they completely snap out. We changed all the instances of the term repair for stability. Now we mention that filamin provides stability to the z-disc.

For example, “All these experimental data have led to the hypothesis that filamin senses mechanical damage at the Z-disc where it coordinates a compensatory stabilization mechanism that prevents the Z-disc from rupturing.”

Lines 88, 90, 348, 378, 380, 381, and 382

Minor Points:

1. Abstract: “either by providing elastic support or through signaling.” Perhaps this should be: “by providing elastic support and/or through signaling.”

Changed

2. Introduction: “Striated muscles are made of myofibrils and repeated sarcomeres.” There are other constituents as well, e.g., mitochondria, nuclei.

Changed

3. Introduction: Confusing sentence should be reordered: “During this process, some Z-disc proteins accumulate at the lesions and are thought to mediate Z-disc repair, including Filamin, Xin, Hsp70, and αB-crystallin [7][8][9].” Change to: “During this process, some Z-disc proteins, including Filamin, Xin, Hsp70, and αB-crystallin [7][8][9], accumulate at the lesions and are thought to mediate Z-disc repair.”
Changed.

4. Results: “Because of the suspected role of filamin in muscle maintenance, we used 15 day old flies”. Authors need to explain the connection between maintenance and the age of the flies, e.g., 15 days is well after myofibrils assembly is complete.

The Nick Brown group showed that filamin mutants stop flying well at 15 days after emergence (Green H. 2018 *eLife* **7**:e35783.) . We added the following sentences. “Filamin myopathy patients show late-onset muscle function loss, indicating filamin's role in muscle maintenance. In Drosophila, filamin mutants fly initially but lose flight ability around day 15. Because of the suspected role of filamin in muscle maintenance, we used 15-day-old flies.”

5. Figure 1D legend: ‘big’ and ‘enlarged’ need not both be used to describe the phenotype.

Changed to “enlarged”

6. Line 241 statement is unclear: We noted that the appearance of ruptured myofibrils decreased in flies prevented from flying but the ratio of frayed myofibrils remained constant between flying and non-flying animals
changed to: We observed a reduction in the occurrence of ruptured myofibrils in flies that were restricted from flying. However, the proportion of frayed myofibrils remained consistent between flying and non-flying flies.

7. Figure 4: Mention that 14-21 mutant myofibrils are not shown. Do the flies mentioned in this figure fly?
All mutants except *filamin ^open^* can fly at reduced levels compared to control in the first week but fall dramatically during the second week.

8. Line 356: Is there a rationale for choosing r > 0.65 as the threshold for synchronous wing movement?

The standard value is r > 0.5. At that value, all the wingbeats from the control flies (Oregon Red and filamin wildtype flies) are synchronized. We raised the threshold value to >0.65 so that only the majority but not all of the control flies would be marked as synchronized. We agree it seems unconventional, and we modified the plot to a threshold of r > 0.5. We changed the plot showing the percentage of synchronized flights in Figure 7 accordingly.

9. Line 484: “we expect our domains to help interrogate filamin functions in other tissues”. Unclear what “our domains” refers to here.

Corrected to “our mutants”

10. Figure S1 Legend. The title is confusing perhaps instead of “contractile damage”, use “damage caused during muscle contraction”.

Changed

**Reviewer #2: General comments:**
This paper presents a well done and interesting analysis of how mutations in the Ig-like domains of filamin affect myofibril structure in the Drosophila IFMs. The authors make use of previously published filamin-GFP mutants that affect the ability of the mechanosensor region (MSR) to operate, presumably in response to mechanical forces. Two different phenotypes are observed in open or closed filamin mutants: ruptured myofibrils or enlarged Z-discs. Further genomic engineering approaches to generate different C-terminal domain deletions narrowed down the Ig 14 domain of filamin as essential for anchoring actin filament at Z discs. The authors also show that muscle contraction is required to induce Z-disc rupture, thereby confirming previous reports that the MSR domain of filamin is required for force transmission. In this sense, the current manuscript largely confirms previous findings rather than uncovering new mechanisms that contribute to filamin function.


ESSENTIAL COMMENTS
• The authors state that Z-discs are enlarged in open filamin mutants. This is indeed true for the Z-disc protein Zasp52 and actin. Are other Z-disc proteins also enriched to substantiate this statement? Or is this recruitment specific to Zasp52?

We propose that the aggregates observed in the filamin open mutants are enlarged Z-discs based on three aspects 1) their position in the myofibrils, between two M-lines. The presence of Zasp52, a typical Z-disc marker, the accumulation of actin, and the presence of filamin, also typically at the Z-disc. We haven’t used any other Z-disc marker. The filamin mutants we made, carry a C-terminal GFP, which is not compatible with the use of the Z-disc GFP protein traps that we and others have described.

Also, the localization of Zasp52 and F-actin are different (see panel 1D and others) as actin seems to be broader. What is the significance of this?

In normal sarcomeres, the actin filaments are anchored to the Z-disc, but these filaments extend until almost to the middle of the sarcomere. Only one small part of the actin filaments is locked into the Z-disc, the most part of the filaments are outside the Z-disc A similar mechanism may explain why actin staining is broader in enlarged Z-discs.

• Lines 82-83 in the last paragraph of the introduction reads ‘We found that the Ig domain pairs 16-17 and 18-19 are required for Z-disc repair.’ This seems to be an overstatement as there are no experiments looking at repair. This statement should be supported with experimental evidence or removed.

We agree that 'repair' is not the appropriate term. We have replaced all instances where we used the term 'repair' with 'stability' (See details in our response to reviewer 1).

• The authors have previously shown that 4 isoforms of filament are present in the Drosophila thoraces (PMID: 28732005). There is no discussion of which isoforms are tagged in the current GFP constructs. How do these additional isoforms affect the interpretation of filamin mutants in the present manuscript since the protein is known to dimerize? Moreover, how do the authors reconcile the specific Z-disc staining of Cher-FLAG (PMID: 28732005) vs the partial Z-disc plus cytoplasmic localization of filamin in Figure 6? Is this an isoform issue?

The description is available in Huelsman 2016 PMID: 27597179. We added the following details in the methods and the results sections: The transcription start site for the cher90 isoform has been omitted from the rescue construct, resulting in the absence of the small cher90 isoform. Since GFP is positioned at the C-terminus, all other isoforms are tagged.

The localization pattern of filamin we report now closely resembles the one we reported in 2017 using other GFP protein trap lines (PMID: 28732005). However, there is a slight difference between the localization observed with UAS-cher90-Flag used in 2017 and that of the protein trap lines, including the new GFP alleles we report here. Specifically, the UAS-cher90-flag shows stronger Z-disc accumulation, whereas the protein trap lines exhibit cytoplasmic localization as well. The accumulation of cher90-Flag at the Z-disc might be an artifact of the overexpression system. Importantly, the UAS-cher90-Flag isoform lacks the N-terminal half of the protein, which may result in an abnormal localization pattern.

• How many copies of filamin-GFP mutants are in used in Figures 1-5? Figure 6 specifically mentions homozygous filamin, but it is not clear in the other figures. Obviously filamin-GFP mutants/Df give rise to adults, but is there partial lethality associated with any of the mutants. It seems like this is a possibility considering the importance of filamin during development and the severe consequences upon manipulation of the Ig domains.

We've included a supplementary data file with the complete genotypes of the flies used for each figure.

While there's some larval lethality linked to filamin mutants, we haven't investigated this phenotype in detail. However, it's probable that this lethality is connected to filamin expression in larval body wall muscles, which have high levels of filamin expression and exhibit a strong phenotype when filamin is depleted by RNAi (PMID: 25715399 and PMID: 32320396).

• Quantitation of the phenotypes in Figure 1 should be included. It is not clear how prevalent these defects are (in 5% of myofibrils? 50%). Maybe a low mag picture would help clarify this issue. More information also needs to be included concerning quantification and statistics. The authors could include this in the methods section. Also include sample size for all graphs. Maybe this is included in Data S2, but this is not mentioned anywhere in the text.

We have incorporated the percentage of affected myofibrils alongside the images in Figure 1. For a more comprehensive representation, a detailed plot is provided in Figure 2, illustrating the phenotypes in flying versus non-flying animals. Furthermore, we included the sample size information in all plots. Additionally, in the Methods section, we now provide details about the number of muscles, their selection process, and specifics about the statistical methods employed.

• The conclusion in panel 2D (enlarged Z-discs are not affected in filamin open mutants) is not convincing as shown. While it is true that the flying vs non-flying appear may not reach statistical significance levels, there is a substantial difference/trend between these two data sets that needs to be clarified. Maybe more sample size or a different type of analysis is required?

Also please clarify what exactly the y-axis (ratio of affaected myofibrils) indicates. Ratio of affected to unaffected? Why not plot this as % affected?

In all plots the ration is the ratio of affected myofibrils over total number of counted myofibrils. We added text in all figure legends that have plots of affected myofibrils in them.

• The Western blots in Figure 4C should include a loading control as the levels of filamin are not equal in different mutants. Are some filamin mutants less stable or degraded at higher levels? Do the lower levels of protein correspond to phenotypes?

We have included loading controls in the form of Ponceau staining on the blots in Figure 4C, which is now presented as Figure S4. Unfortunately, the quality of our blots does not allow making definitive statements about the varying levels in the mutants. Filamin poses a challenge for western blotting due to its low abundance. Nevertheless, we conducted detailed measurements using confocal images to assess GFP intensities of all mutants at the Z-disc (New panels in Figure 6). These measurements suggest that, while some mutants exhibit decreased levels at the Z-discs, others do not, and intriguingly, both groups display ruptured myofibril phenotypes. Consequently, the ruptured phenotype cannot be solely explained by the decreased amount of filamin at the Z-disc.

• Line 237, “Finally, we tested filamins24-attp, the original landing mutant, which removes the entire region starting from Ig 14 and lacks a stop codon. Rupturing Z-discs and fraying myofibrils are common in the filamins24-attp mutant muscles.” There is no picture in Figure 4 to support this sentence.

We added the phenotype of filamin s24-attp mutants as Figure S3.

• There is no control muscle picture in Figure 4. It is not clear why some mutants are repeated multiple times in Figure 4. Is this to show different phenotypes? The graphs in panels F-H contain no statistics or sample size information. Also lines 245-6 seems like an overstatement without additional data. Please modify.

We added a control picture in Figure 4. We added sample size and p values to all the plots. We have removed the statement in line 245-6

• Lines 275-7. The mutations in the main text do not match the figure panels, so please check. IG 16-17 and IG 18-19 are in C and D of Figure 5. Also please include quantitation of filamin-closed as it is mentioned to be the same (line 275).
• Supplemental Figure S1 should be included as primary data.
• The introduction discusses the recruitment of z-disc proteins and the repair of Z-discs associated with Xin, Hsp70 and CryAB. Does filamin interact with these proteins or has an association to cause muscle defects? If any of these ideas have merit there could be some discussion of them. There is an extensive discussion of specific possibilities, but a few general possibilities could be presented too.

OTHER COMMENTS
• Lines 173-4 state that filamin open mutants are flightless but closed are not. This data should be shown here or this statement should be moved elsewhere.

We added flight ability data to figure 1.

• Line 217 is the first mention of the S24 mutation in the body of the text, while the explanation of the mutation is mentioned in the Fig3 legend. Maybe the introduction of S24 is better understood and easier to read in the main text.
• Line 220 – removed ‘well-defined’ as it does not add anything.

Removed.

• Lines 235-236, please clearly identify which panels in Fig4 are being discussed. Also, in line 236, the filamin mutation appears mislabeled. There is no Ig14-21-GFP.

C=added

• Line 237 is confusing. Is this s24-attp mutant the same as attB (integrase landing site from Figure 3A, LINE 222)?

Yes, it is the same, we have corrected all instances into *filamin^s24-attp^*

• Line 312 and lines 314-315 – please include panel letters.

We added panel letters to all references to the main figures.

• Line 351, Incomplete brackets. Figure S1C and S1A, C and A look the same, please specify in the figure legend that 22 is Filamin Δ22-GFP.

Added the brackets. S1c and S1a look similar but they are sightly different, they are not duplicates. Modified the figure legend

**Reviewer #3:**

In this manuscript, the authors investigate the role of Filamin, a mechanosensitive actin-crosslinking protein located at the Z-disc, crucial for maintaining myofibril stability in a muscle context. This study utilizes Drosophila indirect flight muscles (IFMs) and extensively explores the consequences of seven novel mutations on both muscle structure and muscle function. The novel Filamin mutations are located in the C-terminal region, encompassing five that affect the mechanosensitive region (MSR) (Ig14-19), one that affects dimerization (Ig22) and an additional mutation adjacent the MSR (Ig20-21). By analyzing the impact of these different mutations on IFM myofibrils, the authors identify three phenotypes (i.e.: Z-disc rupture, myofibril fraying and enlarged Z-discs) and provide insights to the domains crucial for each phenomenon. Additionally, they demonstrate the necessity of contraction for the development of these phenotypes. Functionally, they reveal asynchronous wing movements in the novel Filamin mutants, indicating compromised muscle force generation due to structurally aberrant myofibrils. The findings not only suggest a conserved role for Filamin in mechanosensing during muscle maintenance but also offer new tools for the study of Filamins through the seven novel mutations. This comprehensive analysis contributes to the understanding of the molecular mechanisms underpinning Filamin’s role in muscle biology, largely unknown to date.

There are several concerns with this manuscript, however.

Data issues
1. Please clarify whether the structural description of Filamin in line 58 is for human filamin, Cheerio, or both. How are the three Filamin genes in human (a,b,c) different from one another? How does Cheerio correspond to these forms? Does Cheerio represent a singular form or encompass all three? This information should be included in the introduction as this is essential for understanding the relevance of the research findings to human disease.

Drosophila has a single filamin gene, while vertebrates possess three. The primary distinction among the vertebrate filamin proteins lies in their expression patterns. Structurally, filamin-a, -b, and -c closely resemble each other, each containing 2 CH actin-binding domains and 24 Ig domains, with the last domain facilitating dimerization. In contrast, Drosophila filamin, encoded by the cheerio gene, features 2 CH actin-binding domains and 22 Ig domains, with the final domain responsible for mediating dimerization.

We have added clarification text.

2. Lines 64-68: it would strengthen the manuscript if a schematic depicting Filamin organization in the sarcomere/myofibril (as described) is shown. This diagram would be particularly helpful to understand how myofibril “fraying” and “fracture” happen and to understand statements such as “actin detachment parallel and perpendicular to the myofibril” (line 245). The schematic could also include information in lines 69-73.

We added three panels to figure 1 depicting the structure of a sarcomere with the localization of filamin highlighted, a cartoon model of filamin at the Z-disc, and a cartoon of the MSR region from the AlphaFold structure model.

3. Could the actin filament dissociation phenotype also be involved with generating the Z-disc rupture (e.g.: if there was not an even loss of actin filaments in a specific region)?

We believe that the fraying of actin filaments does not play a significant role in the rupturing of the Z-discs for two primary reasons. Firstly, observations of both Filamin closed and filamin delta 22 mutants have shown instances of ruptured Z-discs despite the absence of actin fraying. Secondly, the proportion of ruptured discs does not exhibit a notable increase in mutants displaying fraying compared to those with closed or delta 22 mutations. If actin fraying were to indeed enhance the likelihood of rupturing, we would expect to see a clear escalation in the frequency of ruptures, which is not the case.

4. The methods indicate that the behavioral assays were done at one week (line 133). However, the IF analyses were performed at day 15 (lines 130 and 165). Please provide an explanation of why the difference in timing. Also could the authors please comment on the following: Was there a phenotype any earlier than one week? Are these phenotypes cumulative (i.e.: if one performed a time course, would the flies accumulate more fragmented myofibrils or more enlarged Z-discs? Or, alternatively, is the rate always the same?)?

We believe that the rupture phenotypes are cumulative, and thus, we standardized the analysis. Filamin mutants can fly for the first couple of days, roughly a week, and then experience a rapid decline. Filamin myopathies have a late onset. The reason for conducting confocal microscopy after week 2 was to allow time for muscle defects to become apparent. As for the flight assays, we performed them during week 1 rather than week 2 because mutants cease flying after the first week. We also added clarifying text in several parts of the manuscript.

5. Were male and female flies analyzed in the structure and function assays? Are there sex-specific phenotypes?

Only female flies were used. In our previous studies, we utilized the Act88F-Gal4 line, which resides on the X chromosome and exhibits differential expression between males and females (Gonzalez-Morales N. et al 2019. eLife). Consequently, we decided to concentrate on female flies. Although we no longer employ the Act88F-Gal4 driver line in this study, we maintain consistency by exclusively utilizing female flies.

6. Please include the cheerio RNAi phenotype from reference 14, which showed frayed and ruptured myofibrils. The authors discuss these flies and the specific phenotypes, but they are not included for the reader. Flight wing movement data would also be helpful to compare with the other genotypes.

We included a picture of Mef2-Gal4, UAS-filamin-RNAi as figure S1. However, these flies are entirely flightless.

7. On line 165, the text says the control line Filamin WT-GFP does not show any muscle defects. However, upon looking at the image, the sarcomere Y-axis length seems a bit shorter than for filamin WT. Could the authors please clarify? If this is an artifact of the zoom, please correct so that the image size is representative.
There is some variation in the sarcomere height, as depicted in the images we used. We replaced the figure of *filamin ^wt^* with one that matches the size of the *filamin ^wt-gfp^* figure.

8. CRITICAL : For all figures depicting muscle structure and/or ratios:
a. Please explain in detail how the analyses and the statistics were performed (i.e.: Were entire IFMs scanned for rupture, fraying and/or Z-disc enlargement? Were areas of muscle selected at random or the authors followed specific criteria? How many images were analyzed in each experiment? How many replicates were performed?).

We selected areas at random in each muscle and at least 10 muscles were analyzed per condition. We have added details to the materials and methods

b. Please provide statistical tests and p-values in all comparisons made.

We now provide statistical test in the plots for all the comparisons we made.

c. Please also explain in detail how ratios were calculated (i.e.: how was the total count of myofibrils determined? If the count is based on just the number of myofibrils that fit in one image, address the potential variation in myofibril numbers per image by keeping numbers consistent. For instance, each image appears to accommodate around 4-5 myofibers, if one images only 3 or 4 myofibrils –as in panel B-nonflying and panel A-flying, respectively, that results an artificial 20-25% fluctuation of the ratios.

We apologize for the lack of details regarding the calculation of affected myofibrils. The images in all figures are cropped from larger raw confocal images to enhance visibility of the desired phenotype. To calculate the ratios of affected myofibrils, we utilized uncropped images, each containing approximately 25 myofibrils. There may be slight variations in the total number of myofibrils, typically within a range of 1 or 2 myofibrils.

9. Do any of the filamin open-GFP or closed-GFP show frayed myofibrils? Please make this explicit in the text, as this is a phenotype is shown several times in the seven novel mutants. If fraying is found, also provide IF panels with respective quantifications. Please provide an explanation as to why or why not the fraying phenotype is found or not in the open and close filamins in the discussion.

The open and closed mutants do not exhibit the fraying phenotype at all. We believe Ig14 is responsible for maintaining the connection of actin filaments to the Z-disc, in addition to the N-terminal CH domains. Notably, the open and closed mutants do not impact Ig14 or the CH domains in any way. In the results section, we have added the following sentence addressing this point. “In contrast to the large deletion mutants, neither the open nor the closed mutants resulted in frayed myofibrils, suggesting that the mechanical switch of the MSR is not required to prevent actin filament fraying.”

10. In Figure 3, please explicitly mark the location of the MSR in panel B (e.g.: show the Ig colors with 50% transparency or show different Ig colors). While it is mentioned in the text, visually indicating it on the figure will enhance accessibility and comprehension, particularly for readers who are not familiar with Filamins. Additionally, it would be helpful to color code the IG domains that are expected to interact with one another based on the literature.

We acknowledge that the colors used in Figure 3 were not ideal. In response to the suggestions, we have recolored Figure 3. The exons coding for the MSR are now colored in red, and we have added text to emphasize their presence. Furthermore, we changed the colors of the Ig domains removed in each deletion to red for the MSR ones and purple for Ig domains 20-22. Additionally, we included a small cartoon illustrating the positions of the Ig domains 14-22 in a normal filamin

11. In Figure 3C, please provide better image for left panel, particularly of Ig18-19:GFP del and Ig16-17:GFP del, as the text in lines 227-229 indicate only one band of the correct molecular weight and the panel shows another band at 185 kDa. Could the authors show the image of their blots with the loading control so that the reader could get a sense of how much expression is found for each line.

We've included the original blots and the Ponceau staining in the supplementary data. Unfortunately, we don't have higher quality blots available. Filamin is a high molecular weight protein that is not very abundant relative to other myofibril proteins. These conditions make blotting filamin particularly challenging.

12. Please add ruptured and frayed myofibril phenotypes of original landing mutant (s24-attp) in Figure 4 and described in lines 238-289.

We have added two examples of the phenotype caused by the s24 landing site mutant in Figure S5.

13. For the text in the results accompanying Figure 4, please explicitly state in the text whether fraying and ruptured myofibrils were observed in the same muscle or even the same myofibril or whether each phenotype was mutually exclusive to the same muscle or myofiber.
We have added the following “Fraying and ruptured myofibrils were observed in the same muscle or even the same myofibril “and we have added two examples of myofibrils that are both ruptured and frayed in Figure S1.

14. In line 278 the authors mention that enlarged Z-discs have identical sizes, as quantified in S2. Are there panels of Zasp staining for Figure 6? If available, please add the individual grayscale panels alongside the individual actin grayscale panels. We acknowledge the use of a different stain, but please address the observed size difference in the enlarged Z-disc in Figure 6D compared to the measurements shown in Figure S2 panel C.

Panels A to F of Figure 6 and Figure S4 (previously S2) have phalloidin as a counterstain to label actin filaments. We prefer using Zasp52 to measure the Z-disc because of the low background and its specificity to the Z-discs. Actin and filamin are not specific to Z-discs. Figure S4 shows that the size of the enlarged discs is similar between the open and the Δ20-21 mutants. However, the composition of the enlarged discs is not homogeneous. The filamin signal is broader than Zasp52. The reason behind this difference is not clear. Normal Z-discs are not homogeneous; some proteins are exclusive to the Z-disc periphery, like filamin, while Zasp52 and others are specific to the center of the disc. We speculate that enlarged Z-discs maintain the layered organization of the Z-discs. We added the grayscale images of Zasp52 and actin in Figure S4.

15. CRITICAL: Please indicate the full genotype in ALL figure panels (e.g.: filaminΔ20-21:GFP/Df(3R)Ex6176 vs filaminΔ20-21:GFP/filaminΔ20-21:GFP) with an explanation for choosing one genotype over another in each case. Furthermore, note that in many cases, the wrong nomenclature is used (e.g.: “filamin-WT” in Fig 6A and Fig7D is used, instead of the correct form “filamin-WT:GFP”); there is a “filamin-WT” in Figure 1, which makes it confusing. Please be rigorous.

We apologize for the mistakes. We have made a supplementary data word file that contains the full genotypes used in all the figures.

16. Do mutants over deficiencies show the same phenotype (both structurally and functionally) as homozygous mutants?

Yes. We used them over a deficiency background to add a Zasp52-mCherry allele. We used homozygotes not over a deficiency) in figure 6. Similar phenotypes were observed.

17. Could the authors comment on where in the myofibrils these phenotypes are found (e.g.: near the MTJ? towards the center of the muscle?) Also, given that these flies (except the filamin-open) are able to fly, could the authors please discuss how well the flies fly? Are there differences between the genotypes? For example, the authors could use the assay described in this paper to quantify differences: Babcock, D and Ganetzky, B; 2014

The phenotypes are found everywhere in the muscles, to be consistent, we typically image the center of the muscles. We added a line in the methods section.

We added a measurement of flight ability in filamin wt closed, and open.

18. Please provide filamin signal quantification of the filamin re-localization phenomenon from adjacent Z-discs to the ruptured Z-disc described in lines 306-310 and 314.

We added a measurement of the re-localization phenomenon in *filamin ^open^* and is now presented in fig 6.

19. Please make the red asterisks larger in Figure 6. Done
20. Line 356, why is the threshold for synchronous movements r > 0.65? In Fig 7C and D the recordings show r=0.9. While we acknowledge only 5 milliseconds recordings are shown, please provide a representative image of the 1-second recording. A similar issue is found for filamin in a closed state, panel G indicates an r=0.3, however panel L shows approximately 70% of synchronized movements.

Originally, we used r > 0.65 because that is the value at which even some of the control flies have unsynchronized movements. However, we agree that r > 0.5 is more appropriate since it is the commonly used value for correlation statistics. We have changed the measurements using r > 0.5 as a threshold. We have added 1-second recordings in a new Supplementary figure (Fig S5).

21. Please provide a summary table indicating with ALL the genotypes and phenotypes (structural and functional) in the different mutants analyzed (RNAi, open and closed, novel mutants, Ig14-19 deletion) would strengthen the manuscript.

We have made a supplementary data word file that contains the full genotypes used in all the figures. Text S1 also contains a summary table of the myofibril and flight phenotypes.

22. Lines 424-427 say enlarged Z-discs are evidence of myofibril repair. However, the authors should revise the statement and consider several possible scenarios: enlarged Z-discs may potentially represent the first step of damaged myofibrils as opposed to the last phase. Another aspect to consider is the possibility of filamin mislocalization, potentially influenced by interactions with other proteins. Did the authors test other Z-disc proteins? An interesting experiment that would bolster the authors’ conclusions would be to selectively induce damage in specific muscles in filamin-GFP adults and track those over time. This would allow the authors to address whether Z-disc enlargement precedes recovery in the stereotypical fashion described (line 450 onwards), or if those enlarged Z-discs merely are indicators of myofibril damage.

Selectively induced muscle damage and tracking the repair using filamin-GFP or other fluorescent proteins is a great experiment that we are actively exploring. However, the experiment is technically very challenging. Flies cannot be motivated to exercise like humans or even mice. While you can encourage them to move more than usual, for example, by exploiting negative geotaxis behavior with a constantly rotating tube (for examples see Ding M. 2022 doi: 10.3389/fcell.2022.966531), this method does not induce muscle damage to our knowledge. Physically inducing muscle damage using a needle or a blade will trigger a different type of damage that is not contraction dependent. In short, as much as we would like to conduct the suggested experiment, we don’t currently have a way to do it using the genetic tools at our disposal.

We agree that the usage of the term repair is misleading and is based only on the muscle phenotypes of filamin constitutively open and closed mutants. In addition, we don’t know how if at all repair happens in the IFM. We envision the repair as a system that “glues” the damaged and about-to-break Z-discs before they completely snap out. We changed all the instances of the term repair for stability. Now we mention that filamin provides stability to the z-disc.

For example, “All these experimental data have led to the hypothesis that filamin senses mechanical damage at the Z-disc where it coordinates a compensatory stabilization mechanism that prevents the Z-disc from rupturing.”

23. In figure S1, please provide structural IF panels accompanying the quantifications.
Confocal images added

24. In Figure S2 panel B, could the authors comment on the nature of the long and thin GFP-labelled structures on the top-left and bottom-right areas of the enlarged Z-disk? Are those common to all enlarged Z-discs for that particular genotype? How does it compare across different genotypes?
These structures are actin-containing formations, and both filamin open and delta2021 mutants exhibit them. While not all enlarged Z-discs prominently display these structures, they are commonly observed. They are present in both the open and the D20-21 mutants. Currently, we do not fully understand why they assume this particular shape. One possible explanation is that the space between myofibrils is occupied by mitochondria, leading the enlarged discs to grow parallel to the fibrils. We want to explore them further, but doing so requires live imaging which we have not managed to do in the indirect flight muscles.

Text issues

25. Please revise lines 51-62 as they are repetitive (Structure, then human isoforms, then fly isoform, then structure again).

26. Revise line 147, encouraging “flies to fly by blowing air at them” was already mentioned in line 137.
removed second instance

27. Please provide in the text the evidence that the original mutations “lock” Filamin in an open or closed state? Is the Filamin closed state inactive or just not tension responsive? Could this state have other functions?

It requires stronger pulling forces to open

28. Line 266, “ruptured” is said twice.

removed second instance

29. Line 275, “Figure 5B and C” should be “Figure 5C and D”. changed

30. In line 277, did the authors mean Figure 5E instead of 5D? changed

31. Line 281 reads “we analyzed these mutants over a deficiency background…”, this is already mentioned in line 161 and line 290. Please revise, as the wording is very confusing; for example, line 280 and line 353 (wing oscillation experiments) are in homozygotes.

We removed the sentence as it was already mentioned before.

32. Line 351, remove parenthesis at the end of line. Corrected
33. Line 381, “localization” instead of “localisation”. Changed

34. In line 435 and 436, “mechanical” repeated twice. Removed the first instance

35. Line 510, “thefilamin” should be “the filamin”. Corrected

**Have all data underlying the figures and results presented in the manuscript been provided?**
Large-scale datasets should be made available via a public repository as described in the *PLOS Genetics* [data availability policy](http://journals.plos.org/plosgenetics/s/data-availability), and numerical data that underlies graphs or summary statistics should be provided in spreadsheet form as supporting information.

Reviewer #1: Yes

Reviewer #2: Yes

Reviewer #3: Yes

PLOS authors have the option to publish the peer review history of their article ([what does this mean?](https://journals.plos.org/plosgenetics/s/editorial-and-peer-review-process#loc-peer-review-history)). If published, this will include your full peer review and any attached files.


If you choose “no”, your identity will remain anonymous but your review may still be made public.


**Do you want your identity to be public for this peer review?** For information about this choice, including consent withdrawal, please see our [Privacy Policy](https://www.plos.org/privacy-policy).

Reviewer #1: **Yes:** Sanford I. Bernstein

Reviewer #2: No

Reviewer #3: No
