# Supplementary material for: Filamin protects myofibrils from contractile damage through changes in its mechanosensory region
Source: PLoS Genet. 2024 Jun 21;20(6):e1011101. doi: 10.1371/journal.pgen.1011101 (PMC11221683; doi:10.1371/journal.pgen.1011101)
Supplement: Attachment — Submitted filename: Response to reviewers 2.docx [file pgen.1011101.s010.docx]

Response to Reviewers

**Reviewer #1:** The authors have done an excellent job in responding to my comments and I have only one minor concern. The use of the term "heterozygote" to refer to a mutation over a deficiency may be confusing to the reader. I suggest including something like: "heterozygote (mutation/deficiency)" or "hemizygote" to better clarify this.

Thank you. We have checked that all the references to a heterozygote condition correspond to a mutant/+. Heterozygotes were only used in Figure 1J. In the figure legends that include mutant/ deficiency conditions, we added the following “Filamin mutants were analyzed over the *Df(3R)Ex6176* deletion mutant”.

**Reviewer #2:** In many aspects the manuscript is improved. Below are some items that need another look or were not addressed by the authors in their response.

(1) The genotypes in S3 Data do not match the panels in Figure 1, Figure 3, or Figure 7.

We apologize, some of the panel letters in S3 Data were mixed up. We have corrected them.

(2) The response to reviewer states that ‘We have incorporated the percentage of affected myofibrils alongside the images in Figure 1.’ That data is not present. The only graphs in Figure 1 are flight data.

The percentage of affected myofibrils is written below each genotype in Figure 1. Then, plots containing the number of affected myofibrils for each genotype are present in Figures 2C and D.

(3) Sample size is not included for graphs in Figure 1 or Figure 6. This data is also not present in the Figure legends as requested.

We added the sample size for Figure 1 in the legend and the methods. We added the sample size of Figure 6 in the plot.

(4) Maybe the red text on graphs can be changes to a different color. It is difficult to read as shown.

We changed the red text to blue.

(5) The authors did not provide a response to the following query in the original review-

The conclusion in panel 2D (enlarged Z-discs are not affected in filamin open mutants) is not convincing as shown. While it is true that the flying vs non-flying appear may not reach statistical significance levels, there is a substantial difference/trend between these two data sets that needs to be clarified. Maybe more sample size or a different type of analysis is required?

We changed the title of figure2 legend to “Fig 2 Muscle contractions underlie the rupture defects in *filamin^closed-GFP^* mutants.” Now it doesn’t mention filamin open. In the results text, the conclusion of the experiment in figure 2D now reads: “In contrast, the ratio of enlarged Z-discs in the *filamin^open-GFP^* mutants was only minimally reduced.” We believe that these changes fit better with the changes in phenotype counts.

(6) The authors state ‘We have included loading controls in the form of Ponceau staining on the blots in Figure 4C, which is now presented as Figure S4.’ This not correct as the blots are Fig. S2.

Yes. We changed the previous response to reviewer’s document.

(7) The following statement was not addressed by the authors - It is not clear why some mutants are repeated multiple times in Figure 4. Is this to show different phenotypes?

Some mutants are shown more than once to clearly show both the frayed and the broken myofibril phenotypes.

(8) The authors did not provide responses to the following queries in the original review-

• Lines 275-7. The mutations in the main text do not match the figure panels, so please check. IG 16-17 and IG 18-19 are in C and D of Figure 5. Also please include quantitation of filamin-closed as it is mentioned to be the same (line 275).

We checked and corrected the references for Fig 5 panels in the text. We added a reference to the figure where the quantification of filamin-closed phenotypes is presented.

• Supplemental Figure S1 should be included as primary data.

We do not agree. Fig S1 contains the filamin-RNAi phenotype. This phenotype has already been described extensively by us previously (Fig1 is already data-heavy and adding FigS1 into it will make it harder to read).

• The introduction discusses the recruitment of z-disc proteins and the repair of Z-discs associated with Xin, Hsp70 and CryAB. Does filamin interact with these proteins or has an association to cause muscle defects? If any of these ideas have merit there could be some discussion of them. There is an extensive discussion of specific possibilities, but a few general possibilities could be presented too.

We added a brief mention in the discussion: “We do not know the signaling molecules downstream of filamin opening. However, two possible proteins are Hsp70 and αB-crystallin. They both localize to muscle damage like filamin [7–9], and the Drosophila αB-crystallin physically interacts with filamin in muscles and causes muscle defects when removed [18]. A speculative scenario is that filamin opening recruits αB-crystallin which helps stabilize the Z-disc.”

We do not mention Xin because there is no direct homolog in Drosophila.

**Reviewer #3:** While the revised manuscript is improved, several issues remain and are listed below. In addition, the writing was could be significantly improved.

Abstract:

1. Lines 19-21: Authors write “We made novel filamin mutations affecting the C-terminal region to interrogate the mechanosensitive region and detected two Z-disc phenotypes: dissociation of actin filaments and Z-disc rupture”. However, isn’t the enlarged Z-disc observed in the deletion of Ig20-21 also a phenotype? We acknowledge it is not the MSR region, but that deletion does, indeed, affect the C-terminal region.

We changed the statement accordingly.

2. On line 26, we suggest inserting “stabilizes” before “and counteracts contractile damage at the Z-disc”, as it is a key aspect discussed in the paper.

We changed the statement.

Introduction:

3. Line 48: Authors write “The Z-disc is a thin structure that bisects the sarcomere”. This statement is inaccurate, the Z-disc defines the boundaries of the sarcomere, it does not bisect/divide the sarcomere in two parts.

We corrected the statement.

4. Line 71: Please correct typo “Drosophila” instead of “Drosophia”

We corrected the typo.

5. Line 92: Authors state “enlarged Z-discs, which we interpret as a compensatory mechanism to stabilize the Z-disc and prevent it from breaking”. It is unclear, however, what this a compensatory mechanism is for. Please specify whether this is a compensatory mechanism for eccentric contractions and/or sarcomeric damage and/or both, if that is indeed what authors mean.

We modified the statement into the following. “as a compensatory mechanism for the sarcomere damage that occurs during eccentric contractions to stabilize the Z-disc and prevent it from breaking.”

Methods:

6. Please specify that Ponceau staining was used as a loading control and the specifics regarding that stain.

We added the following details to the method section. “As loading control, we used Ponceau staining (5% glacial acetic acid and 0.1% Ponceau S), followed by washing with 1X Tris-Buffered Saline 0.1% Tween.”

7. In the response to reviewer 3, point 8c, authors indicated that in order to calculate the ratios of affected myofibrils, they used uncropped images each containing approximately 25 myofibrils +/-2. However, in lines 145-146, authors state that to calculate ratios of affected myofibrils, they used uncropped images typically containing 10-15 myofibrils. Please clarify this discrepancy.

We are sorry for the mistake. 10-15 myofibrils are the correct numbers.

Results:

8. Line 197: Add “Fig. S1” next to reference 16.

Added.

9. Overall comment for figures 4, 5, 6 and 7: Please keep a consistent order for the genotypes both in the IF images and corresponding plots. The current inconsistency in the order makes it very confusing to follow, as the order changes within the same figure and across figures.

The microscopy images are arranged to match the order in which they are presented in the result text. The genotypes in the plots are sorted according to their values. E.g., Fig6G shows the fluorescence intensity of the filamin mutants tagged with GFP at the Z-disc. The genotypes are sorted from the one with the highest intensity at the Z-disc (*filamin-WT-GFP*) to the one with the least amount (*filamin-open-GFP*). We believe this sorting makes the plot easier to follow than the alternative.

Figure 1:

10. Indicate in panels A and B that the contractile forces are referring to eccentric contractile forces.

We decided to remove the term "eccentric" throughout the paper to enhance readability.

11. Panel B needs more clarity and specificity. We suggest adding more details (i.e.: indicate where the Z-disc is, and clear labeling N-terminal and C-terminal regions of filamin).

Added N-terminal and C-terminal labelling.

12. Modify colors of panels A and B so that they match the staining from the IFs (i.e.: highlight Z-discs in panel A in magenta instead of green; in panel B, use a different color for filamin, as green can be confusing given the IF stainings from the panels below, which also use green for actin).

We changed the colors.

13. Please describe in the methods how the “Percentage that flew” was assessed. Also, we suggest changing the axis as “Percentage of flying adult Drosophila” for better clarity.

We changed the axis name as suggested. We added the following statement. “For flight assays, 30 flies were released individually from a plastic vial: if they flew upwards, they were marked as a flyer, whereas if they fell or glided to the ground they were marked as a non-flyer.”

Figure 6:

14. Please describe in the methods the specifics on how the GFP intensity quantification from Figure 6 was performed (e.g.: line plots across the Z-disc? Mean gray value within an area? How was the area selected? etc).

We added the following sentence to the methods section. “To measure GFP intensity, we utilized the following ImageJ script, which creates a 250-pixel circle around a selected spot and calculates the average gray value.

macro "Macro enlarge measure [a]" {run("Enlarge...", "enlarge=4 pixel"); run("Measure");}”

Figure 7:

15. Panel D still shows the wrong genotype, please change for WT-GFP.

We corrected the label.

16. Lines 320-322: The statement “The filamin closed-GFP homozygote mutant flies had irregular wing beats with low r values (Fig 7G, r = 0.3), indicating that they struggle to maintain wing movement synchrony” conflicts with Figure 7L, which shows 80% synchrony. This raises an issue in how the parameters (i.e.: synchronicity and correlation) are defined in the text and needs clarification. We understand the Pearson’s correlation coefficient is the indicator of a match between wing left and wing oscillation amplitude, however, it is important to note that even if the oscillations might not have the same amplitude (r=0.3), they might still exhibit high synchronicity (80% of events). Line 315 should be revised to provide better understanding in how to interpret the Pearson’s correlation coefficient, and the text in the results sections should be adjusted accordingly to reflect these interpretations.

The Pearson correlation coefficient measures the linear relationship between two sets of data, specifically in this context, the quantity of reflected light captured by the right and left sensors. It does not evaluate the amplitudes of the data sets. Due to the comparison of the right and left light levels at each time point, minor out-of-phase discrepancies result in diminished correlation coefficients (R values). Figure 7G illustrates an instance where certain wing movements exhibit synchronization, while others do not. Initially, during the first two wing beats, the right wing initiates slightly ahead of the left wing (not synchronized). The following five movements start and finish simultaneously (synchronized). However, in all subsequent movements, the right wing commences prior to the left wing (not synchronized).

We then used a threshold of r > 0.5 to categorize recordings as synchronized (r>0.5) or unsynchronized (r< 0.5). We counted all of them to estimate the penetrance of the phenotype (i.e., number of flying events that are synchronized). Figure 7L shows the penetrance of the asynchrony phenotype in different genetic backgrounds. Control files are always synchronized (100% events) while the mutants are not. Filamin closed mutants have ~20% unsynchronized movements which is more than the 0% the controls have.

We added additional details to the methods and to line 315 of the results.

Figure S3:

17. Please add p-values/stats in panel C, as well as “n” in panels A, B, and C.

Added

S3 Data:

18. Please change the letters to match the panels in Figure 1 (i.e.: the first genotype should correspond to the letter D, and so on, until the letter J).

Done

19. Genotypes denoted in Figure 7 of the Word document do not correspond with the order of notation in the blots, please rearrange Word genotypes in order to keep the same order as in the blots.

Figure 7 does not have blots on it, Figure 3 does. We arranged the genotype names in the S3 Data file to match the order of Figure 3.

20. Edit panel names for Figure 7 in word document to match the figure panel letters.

Done

Have all data underlying the figures and results presented in the manuscript been provided?

Large-scale datasets should be made available via a public repository as described in the PLOS Genetics data availability policy, and numerical data that underlies graphs or summary statistics should be provided in spreadsheet form as supporting information.

We have added an Excel file containing the myofibril microscopy data, the GFP intensity data, and the wing movement correlation data.
